# Supplementary material for: Single-cell transcriptomics identifies master regulators of neurodegeneration in SOD1 ALS iPSC-derived motor neurons
Source: Stem Cell Reports. 2021 Nov 11;16(12):3020–35. doi: 10.1016/j.stemcr.2021.10.010 (PMC8693652; doi:10.1016/j.stemcr.2021.10.010)
Supplement: Document S1. Supplemental Experimental procedures, Figures S1–S5, Tables S1, and S2 [file mmc1.pdf]

Stem Cell Reports, Volume *16*

Supplemental Information

Single-cell transcriptomics identifies master regulators of neurodegeneration in SOD1 ALS iPSC-derived motor neurons

Seema C. Namboori, Patricia Thomas, Ryan Ames, Sophie Hawkins, Lawrence O. Garrett, Craig R.G. Willis, Alessandro Rosa, Lawrence W. Stanton, and Akshay Bhinge

Supplementary methods

Differentiation of iPSC into spinal motor neurons

iPSC were plated as colonies onto matrigel and differentiated by treatment with neuronal differentiation media (DMEM/F12:Neurobasal in a 1:1 ratio, HEPES 10mM, N2 supplement 1%, B27 supplement 1%, L-glutamine 1%, ascorbic acid 5uM, insulin 20ug/ml) supplemented with SB431542 (40uM), CHIR9921 (3uM) and LDN8312 (0.2uM) from day 0 till day 4. Cells were caudalized by treatment with 0.1uM retinoic acid starting at day 2 and ventralized with 1uM purmorphamine starting at day 4 and continued till day 10. At day 10, progenitors were re-plated onto poly-D-lysine/laminin coated wells and differentiated by treating the cells with N2B27 media supplemented with BDNF 20ug/ml, GDNF 10ug/ml and DAPT 10uM. DAPT treatment was stopped at day 14 and neuronal cultures were pulsed with mitomycin at a dose of 10ug/ml for 1 hour to prevent further proliferation of any undifferentiated progenitors. Neuronal cultures were maintained by changing media every other day.

Immunofluorescence

Cells were fixed with 4% paraformaldehyde, permeabilized with ice-cold methanol for 5 minutes and washed with PBS containing 10% serum for 1 hour at room temperature. Cells were incubated with primary antibodies (Table S1) diluted into PBS containing 10% serum and incubated overnight at 4°C. Next day, cells were washed and incubated with Alexa-fluor conjugated secondary antibodies (Molecular probes) for 45 minutes at room temperature and nuclei were stained with Hoechst 33542 (Molecular probes). Images were obtained in an automated fashion on the ImageXpress Pico (Molecular devices). ISL1+ nuclei identification and p-SMAD2 nuclear signal was quantified in an automated fashion using the imageXpress software. Threshold intensities were maintained the same across wells.

Quantitative RT-PCR

Total RNA was extracted with the miRNeasy kit (Qiagen) and reverse transcribed using random hexamers and the HighCapacity reverse transcription system from Applied

Biosystems. Quantitative PCR was performed using the SYBR GREEN PCR Master Mix from Applied Biosystems. The target gene mRNA expression was normalized to the expression of two housekeeping genes (HPRT1 and RPL13), and relative mRNA fold changes were calculated by the ΔΔCt method. Primer sequences are included in Table S2.

MN survival assay

MNs were differentiated from SOD1 E100G and the isogenic control iPSC as described above in 96-well optically clear tissue culture plates. Day 30 cultures were fixed and stained for ISL1 to assess MN counts. A separate plate cultured under the same condition was allowed to proceed till day 44, when it was fixed and stained for ISL1. Nuclei were stained using Hoechst 33342. MN counts at day 40 were compared with day 30 to assess MN survival. We performed three independent differentiations with two technical wells used per replicate. Data from the technical wells was pooled to generate counts for each replicate.

Cell survival assays in response to TGFβ perturbation

We found that the process of immunostaining occasionally resulted in neuronal detachment leading to underestimates of neuronal counts. Hence, cultures that showed neuronal detachment were discarded. We realized that a better approach was to treat live neurons with the cell-permeable nuclear dye Hoechst 33342 and image the same well at different time points. Given that almost 80% of the cells in culture were motor neurons, nuclei counts were expected to closely approximate MN counts. Neuronal cultures at day 30 were treated with Hoechst 33542 (0.25 ug/ml) for 45 minutes and media was replaced with standard neuronal culture media as described above. Nuclear stained cultures were analysed by live imaging in the DAPI channel at day 30 and again at day 40. This allowed us to assess nuclear counts for the same well at the two different time points. Day 40 counts were compared with day 30 counts to assess percentage loss of neurons using 3 independent differentiations with two wells as technical replicates per treatment per differentiation. SB431542 was dissolved in DMSO. Hence, a DMSO only treatment was used as control for the TGFβ inhibition experiments. The final concentration of DMSO was maintained at 0.1%. TGFβ1 was

resuspended in PBS and we used neurons treated with PBS as control. Apoptosis was estimated in independent neuronal cultures using the Promega RealTime Glo apoptosis assay according to the manufacturer's instructions.

Single-cell capture and library preparation

Single cells were captured using standard protocol of C1 single-cell auto prep system (Fluidigm). Two independent differentiations were set up a few days apart. At day 44, differentiated neuronal cultures were dissociated into single cells by Accutase and loaded onto the C1 chip. We used one chip per genotype per differentiation. Chips for each replicate (one for the SOD1 E100G and the other for the isogenic control) were loaded in parallel into two separate machines. We used a total of four chips labelled A, B, C and D. Chips A and B captured the isogenic control MNs while chips C and D captured the SOD1 E100G MNs. Post cell capture, each well of the chip was manually inspected to identify wells bearing single cells. Next, lysis, reverse transcription and PCR amplification of the cDNA was perform in an automated fashion within the C1 instrument. To prepare single-cell libraries, cDNA products from each single cell were harvested from C1 chip followed by concentration and quantification using PicoGreen dsDNA Assay kit. Sequencing libraries were generated using Illumina Nextera XT library preparation kit.

Read processing, mapping and quality control

Fastq files were processed using Salmon with a partial decoy index for human gene annotation GENCODE release 19 (Harrow et al. 2012). Transcript level counts were collapsed to generate read counts per gene using the tximport R package. This yielded 33,000 transcripts across 365 libraries. Only libraries deemed to be single cells were retained for further analysis (332 cells). A gene was deemed to be poorly expressed if it was present in less than 10 cells at a read count threshold of 2. The filtering process yielded 332 single cells and 14774 genes for further analysis. To remove poorly amplified RNA libraries, single cell libraries were subjected to a set of quality control criteria that included: 1) total mapped reads, 2) percentage mapped reads,

3) percentage of mitochondrial reads, 4) number of genes expressed. To identify cells that were outliers, we performed a PCA using these criteria (Fig. S1a). The PC1 vs PC2 map identified cells that were outliers with respect to the number and percentage of mapped reads while PC3 identified cells that displayed low number of expressed genes and high levels of mitochondrial reads (Fig. S1a). Cells deemed as outliers were removed from further analysis. After quality filtering, we retained a total of 323 high quality cells (163 cells for SOD1 and 160 cells for the control). Next, we removed lowly expressed genes (counts < 2) that were expressed in less than 10 cells. In summary, we obtained 323 cells that expressed 14774 genes in total with SOD1 cells expressing on average 7170 genes while the control dataset expressed 7745 genes with the overall distribution of the number of genes being similar between the two datasets (Fig. S1b). Additionally, other quality controls metrics including total number of reads, proportion of mapped reads, proportion of mitochondrial reads, duplicated sequences and GC content were similar across the SOD1 and isogenic control datasets (Fig. S1b). Differences between the isogenic control and ALS datasets for the proportion of mapped reads, mitochondrial reads and duplication rates were deemed significant by a t.test (Fig. S1b). However, the magnitude of the differences was small (0.68 vs 0.73 for the proportion of mapped reads, 21.7% vs 14.5% for the % mitochondrial mapped reads, 54.4% vs 58.8% for the duplication rate). We noted that the isogenic control cells from the replicate 2 displayed significantly higher percentage of mitochondrial mapped reads than replicate 1. Hence, it was deemed appropriate to control for this potential batch effect in the differential expression analysis. Each gene was classified based on whether it was protein coding, long non-coding, pseudogene or small nuclear/nucleolar RNA. We did not find any systematic difference in distribution of the gene classes expressed between the SOD1 and controls datasets (Fig. S1c). Further, principal component analysis using all expressed genes confirmed that our data did not show any batch effects arising from the independent differentiations or the capture plates (Fig. S1d). Finally, the PCA did not reveal any bias towards any specific dataset for the number of mapped reads, number of detected genes or mitochondrial genes (Fig. S1e).

Identifying genes affected by ambient RNA

Single cell dissociation and harvest protocols lead to lysis of cells that release their cellular mRNA into the mixture. This RNA termed "ambient mRNA" is released from dying cells and has been observed in the absence of cell capture in droplet based platforms(Angelidis et al. 2019). The ambient mRNAs typically arise from genes expressed at high levels. We observed that some of the microfluidic chambers that were marked as empty had generated RNA reads that mapped to human transcripts. It is possible that these chambers contained a cell that was missed by the human observer. But we decided to treat transcript counts arising from empty chambers as ambient mRNA. For each plate, we summed up the counts per gene across the empty chambers and estimated counts per million (cpm) reads for each gene. Next, we averaged the cpm values per gene across the two replicates plates for the healthy and ALS samples separately. We marked all genes at a cpm threshold above 100 as being affected by ambient RNA expression. We performed this filtering for the healthy and ALS samples individually to avoid excluding genes that were lowly expressed in either the healthy or the ALS samples. This resulted in a total of 1120 genes being marked as affected by the ambient mRNA. The ambient geneset was excluded from the clustering analysis to identify neural subtypes. Additionally, we excluded these genes from the sorted differentially expressed geneset used in the master regulator analysis (see below). This genelist has been included as Supplementary data file S1.

Identifying classifier genes

Neuron vs glia classifier gene set

We first identified genes differentially expressed between neurons and glia using a recently published gene expression dataset on purified human neurons, astrocytes and oligodendrocytes from frozen brain tissue(Zhang et al. 2016). Genes that displayed a fold change of at least 20 between neurons and astrocytes or oligodendrocytes were included for future analysis. Differential gene expression analysis identified 707 genes as differentially regulated between neurons versus glial cells. Gene ontology using DAVID(Huang da et al. 2009) on the differentially expressed gene set showed enrichment of specific functional

categories related to neuronal physiology. Categories related to neuron development such as GABAergic synapse and postsynaptic cell membrane were enriched in the neuron-activated genes while cell cycle and glial differentiation terms were deemed to be enriched in the downregulated genes confirming that our identified gene set was able to distinguish neurons and glia (Fig. S2a). Out of the 707 genes, 682 genes were expressed in our filtered single cell dataset. This list of genes was termed neuron_vs_nonneuron.

MN vs IN classifier gene set

To identify genesets that can differentiate between MNs and INs, we used the "knowledge matrix" defined previously to classify neurons isolated from embryonic mouse spinal cord into specific subtypes (Delile et al. 2019). This a binary matrix mined from published literature on key genes expressed in specific neuronal subtypes. We updated this matrix to include the following genes that are highly expressed in glial cells and progenitors: S100B, SOX9, PAX6, MKI67 and REST (Supplementary data file S2). In total, the matrix comprised of 52 genes and 14 cell types. Next, we extracted the expression data for the 52 genes from our single cell read counts for the healthy cells and binarized the counts for each gene using K-means clustering. We performed this analysis only on the healthy cells to avoid including any influence of the SOD1 mutation on marker gene expression. Our goal was to use this initial set of 52 genes to extract a wider gene set that can be used to classify the SOD1 and healthy data sets. The expression profile for each gene across the healthy cells was clustered using k=2 that separated the profile into low and high expression clusters. The high expression cluster was used to estimate a threshold for binarizing the gene expression vector (Methods). This resulted in a binary profile for the 52 markers gene for each cell. Each cell was now compared with each of the cell types in the knowledge matrix by estimating a jaccard coefficient (JC), which was used to classify a cell as a MN or IN. Due to the low number of cells, we did not distinguish between IN subtypes at this stage. To validate our classifier, we used it to identify cell types in an external dataset that include gene expression profiles of MNs, INs and progenitors purified using FACS from embryonic mouse spinal cord(Amin et al. 2015). We were able to classify all profiled cell types correctly into three main classes, namely MNs, INs, and non-neuronal cells

(Fig. S2b). We deployed our classifier to identify the top 25 cells (based on the JC) classified as MN or IN in our dataset. Gene expression of markers indicated that the classification was accurate (Fig. S2c). These cells were used to generate a differential expression list that could be used to distinguish MNs from INs based on their expression profile. This set of 600 genes, termed the MN_vs_IN geneset, was combined with our neuron_vs_nonneuron list described above. After filtering for genes affected by ambient RNA expression (Methods), we generated a list of 1060 unique classifier genes.

Differential expression analysis of MN sub-clusters

We noted that cluster 2 in Fig. 1f had lower number of SOD1 MNs compared to the isogenic controls. To ascertain that differences in the membership of SOD1 and control neurons was not driving the observed changes, we performed differential gene expression analysis of clusters 1 and 2 separately. We identified 129 genes as downregulated and 471 genes as upregulated in SOD1 ipMNs in cluster 1 at a p-value threshold of 0.01. Out of the 129 genes, 55 were also downregulated in the full dataset while the 305 genes overlapped between the upregulated genesets (Fig. S2e). Due to the low number of cells in the SOD1 set in cluster 2, we identified only 2 genes as significantly downregulated and 0 genes as upregulated in SOD1 ipMNs at an adjusted p-value threshold of 0.01. Applying a less stringent p-value threshold of 0.1 identified 137 genes as downregulated in SOD1 ipMNs, a number comparable with the 170 genes downregulated using all the ipMNs. Only 14 of the 137 genes overlapped with the downregulated genes identified using the full dataset though the overlap was statistically significant (Fig. S2f). At the p-value threshold of 0.1, only 8 genes were identified as upregulated in the cluster 2 set (Fig. S2f). Next, we performed pathway enrichment analysis of differentially expressed genesets in clusters 1 and 2 using GSEA (Fig. S2g, S2h). GSEA revealed downregulation of gene sets pertaining to mitochondrial gene expression in cluster 1 SOD1 MNs while mitochondrial respiratory transport and oxidative phosphorylation gene sets were downregulated in cluster 2 SOD1 MNs. Lowering the adjusted p-value threshold stringency of the GSEA to 0.1 revealed gene sets pertaining to axon guidance, neuron

projection and anterograde trans-synaptic transmission as downregulated in SOD1 MNs from the cluster 1 dataset but not in the cluster 2 dataset. Both datasets showed upregulation of the cell cycle pathway while terms related to mitosis, nonsense mediated decay and ribosome were upregulated in the cluster 1 MNs (Fig. S2g, S2h). These results indicate that differential membership in cluster 2 was not driving the observed differential expression in the full dataset.

kNN smoothing of single cell read counts

We adapted the algorithm described by Wagner et al(Wagner et al. 2018). Briefly, the read count smoothing algorithm worked as follows: 1) We started with a matrix of N cells X m genes where N=189 and m=14774 2) Count data was variance stabilized by taking the logarithm of the counts after adding 1 for each value in the single cell matrix. This was because, for our dataset, the log transformation worked better than the Freeman Tukey transform used in the original study. 3) Log transformed read counts were quantile normalized. 4) Euclidean distances between the cells were calculated using the first 8 principal components. The threshold of 8 was determined empirically using a scree plot. 4) For each cell, we calculated a weighted average of the read counts per gene between that cell and its k neighbours. The weights for the averaging are assigned as the inverse square root of the Euclidean distance between the cell and its neighbour. This ensured that neighbours that were far away in Euclidean space did not contribute as much to the final smoothened count than the nearest neigbours. The value of k is iteratively increased starting from 1 according to the following equation: $k = \min(2^{step} - 1, kmax)$; kmax = sqrt(N); step = 1:max_steps; max_steps = floor(log2(kmax + 1)); For example, in our study, for N=189, we get kmax=14 and max_steps=3.0. This results in 3 iterations where the value if k equals 1, 3 and 7 in each iteration successively. The final output is a matrix of counts that are quantile normalized and log transformed.

Estimating WGCNA module robustness

We estimated module robustness using a method detailed by Shannon et al(Shannon et al. 2016). The 189 neurons were sampled 50 times with replacement and modules were identified using the smoothened gene expression profiles for each sampling. The number of modules obtained per iteration ranged from 24-33 with a median of 29, which was close to the number obtained using all 189 neurons (26 modules). Each module in the original set of 26 modules identified using all 189 neurons was compared with all modules obtained in each iteration. Similarity between two modules A and B was defined as $(A \cap B)/\min(A, B)$ where $A \cap B$ indicates the number of genes common to A and B, while min(A,B) indicates the size of the smaller module between A and B(Shannon et al. 2016). We retained the maximum similarity score for each of the 26 modules per iteration. The final stability scores for each module were calculated as the average similarity scores across the 50 simulations.

Master regulator analysis

Smoothened counts for 189 neurons were used as input to the ARACNE algorithm to build a transcriptional network for 1137 TFs present in the dataset. TF annotations were obtained from AnimalTFDB. P-values for network edges were estimated from a pooled null distribution using 1000 permutations. Since we were asking ARACNE to evaluate interactions between 1137 TFs and 12550 target genes, a p-value threshold of 5e-8 would result in < 1 (1137*12550*5e-8) false positive edges to be included in the final network. At a p-value threshold of 5e-8, we identified a total of 1,255,493 edges between 1137 TFs and 12550 target genes with an average of 987 targets predicted per TF. The predicted targets of each TF were termed as the regulon. The regulon for each TF was classed as positive or negative based on the Pearson correlations. To identify master regulators, the differential gene expression between SOD1 and control ipMNs (after removing the ambient geneset) was used as a phenotype and sorted from most upregulated to most downregulated. The RTN package was used to conduct a GSEA like analysis to identify whether a TF regulon (positive or negative) was enriched towards one end of the sorted list of differentially expressed genes. P-values were

estimated based on 1000 permutations of the dataset and adjusted using the Benjamini Hochberg method. GO analysis of the regulons was carried out using the anRichment R package.

Analysis of publicly available ALS datasets

Normalized read count data for GSE54409 (human SOD1 A4V iPSC derived MNs purified using flow sorting based on the HB9 reporter)(Kiskinis et al. 2014) were downloaded from the gene expression omnibus(GEO). P-values were estimated by performing a t.test per gene. Read counts were averaged across replicates per gene and log2 transformed. Fold changes were estimated by subtracting the log2 counts for the ALS and isogenic controls: log2(ALS) – log2(control). Microarray expression values for GSE46298 (laser-capture microdissected MNs from spinal tissue obtained from the mouse SOD1 G93A ALS model)(Nardo et al. 2013) and GSE18920 (laser-capture microdissected MNs from spinal tissue obtained from sporadic ALS patients post-mortem)(Rabin et al. 2010) were downloaded from the gene expression omnibus. Expression values were background subtracted, normalized and log transformed using RMA from the affy R package(Gautier et al. 2004). Only genes with median expression values above background were included in the analysis. Probes mapping to the same gene were collapsed. Differential expression analysis was performed using the limma package in R(Ritchie et al. 2015). Differentially expressed genes for GSE76220 (laser-capture microdissected MNs from spinal tissue obtained from sporadic ALS patients post-mortem) (Krach et al. 2018) were obtained from the supplementary data for that study. This study had removed genes activated by the wound healing response by excluding genes with the highest principal component (PC1) eigengene values. We obtained both, the full (all genes) and the filtered (PC1 adjusted genes) datasets. Count data for GSE98920 (VCP R191Q, R155C) was obtained from GEO(Hall et al. 2017) while counts for the bulk C9ORF72 datasets were requested from the authors(Selvaraj et al. 2018). Both sequencing datasets were analyzed using DESeq2. For GSE98288, we only used counts from the control iPSC differentiations. P-

values for all datasets were corrected for multiple hypotheses using the Benjamini Hochberg procedure. Differentially expressed genes identified from our bulk RNA-seq analysis of SOD1 E100G iPSC derived MNs were obtained as described previously(Bhinge et al. 2017).

Count data for GSE140747(Rayon et al. 2019) (motor neuron development gene expression: D0-D15) and GSE98288(Hall et al. 2017; Luisier et al. 2018) (D21, D35 vs iPSC) was downloaded from the gene expression omnibus. We used DESeq2 to generate a list of genes differentially expressed between D0 neuromesodermal progenitors (NMPs) and D15 MNs. This list was sorted such that genes most upregulated in the NMPs were at the top while genes most downregulated in the NMPs were at the bottom. This process was repeated for time points D1 to D8 generating a set of differentially expressed ranked genelists for neural progenitors. By reversing the genelist D7 vs D15, we generated an additional ranked list of genes that had neuron-specific genes at the top of the list. Additionally, we also generated ranked gene expression profiles in immature (D21) and mature (D35) MNs differentiated from healthy iPSCs(Hall et al. 2017; Luisier et al. 2018). To sort genes, each gene was assigned a score = -1*log10(p-value) * sign(fold change). Genes were sorted based on this score in a decreasing manner so that the most significantly up regulated genes were assigned to the top of the list.

Supplementary figures

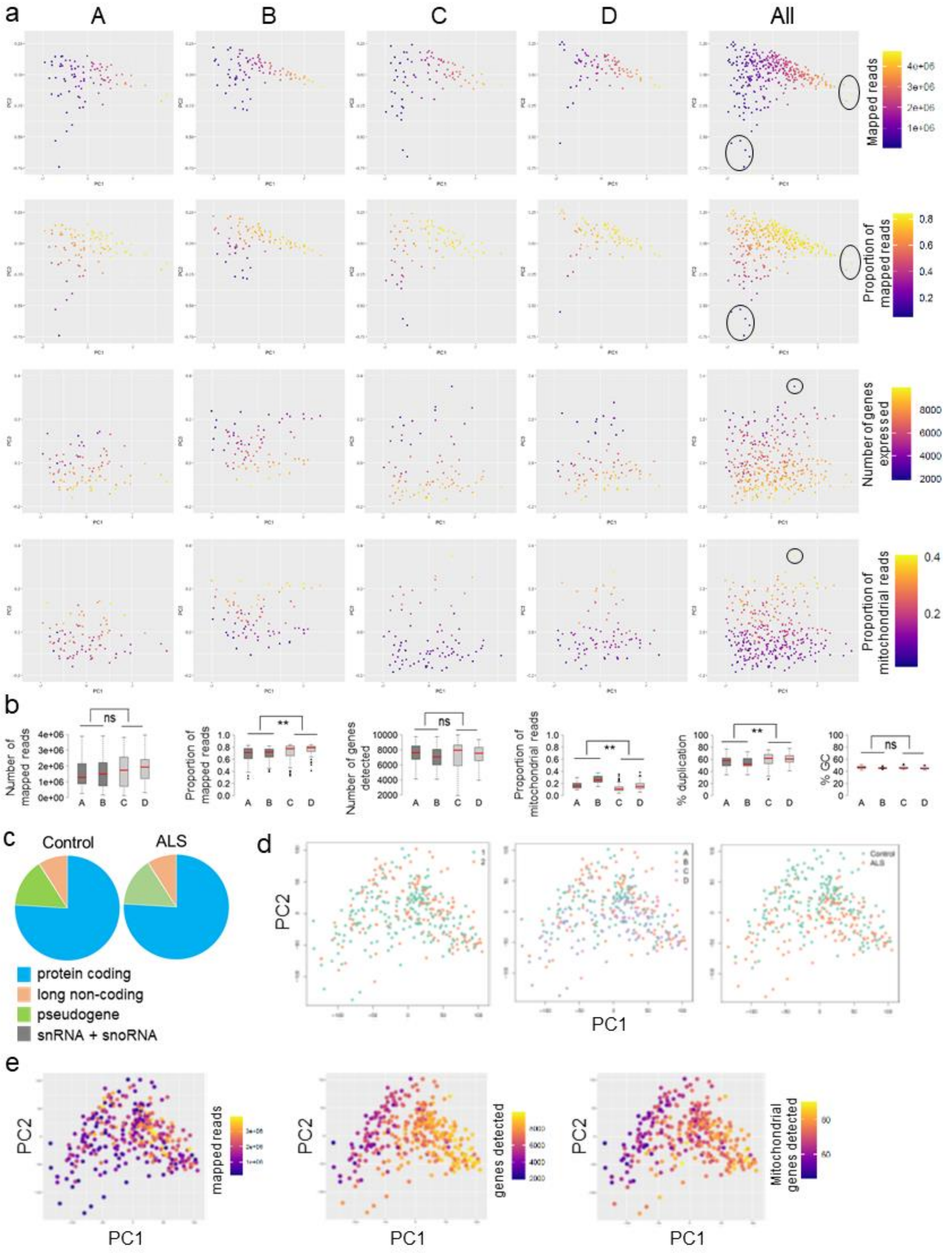

Fig. S1. Quality check of the single cell data

a) PCA plots displaying the different quality metrics used to filter cells. Each dot represents a cell. The black ellipses mark cells classified as outliers. Overall, nine cells were identified as outliers. b) Boxplots showing number of mapped reads, proportion of mapped reads, number of expressed genes, proportion of mitochondrial genes, percentage duplication and GC content in control and SOD1 datasets after removing outlier cells. P-values were estimated using a two-tailed Students's t.test by comparing the isogenic control dataset (cells from plates A, B) with the SOD1 dataset (cells from plates C, D). c) Pie chart showing the distribution of the expressed genes in different classes. d) PCA plots generated using all expressed genes show there is no batch effect between the datasets. Each dot represents a cell. Left panel: cells have been coloured based on the replicates. Middle panel: cells have been coloured based on which C1 fluidigm plate they were captured in. A: Control replicate 1, B: Control replicate 2, C: SOD1 replicate 1, D: SOD1 replicate 2. Right panel: Cells have been coloured based on genotype (i.e, isogenic control or ALS SOD1 E100G). e) PCA plots generated using all expressed genes showing distribution of mapped reads, proportion of mapped reads and number of mitochondrial genes detected.

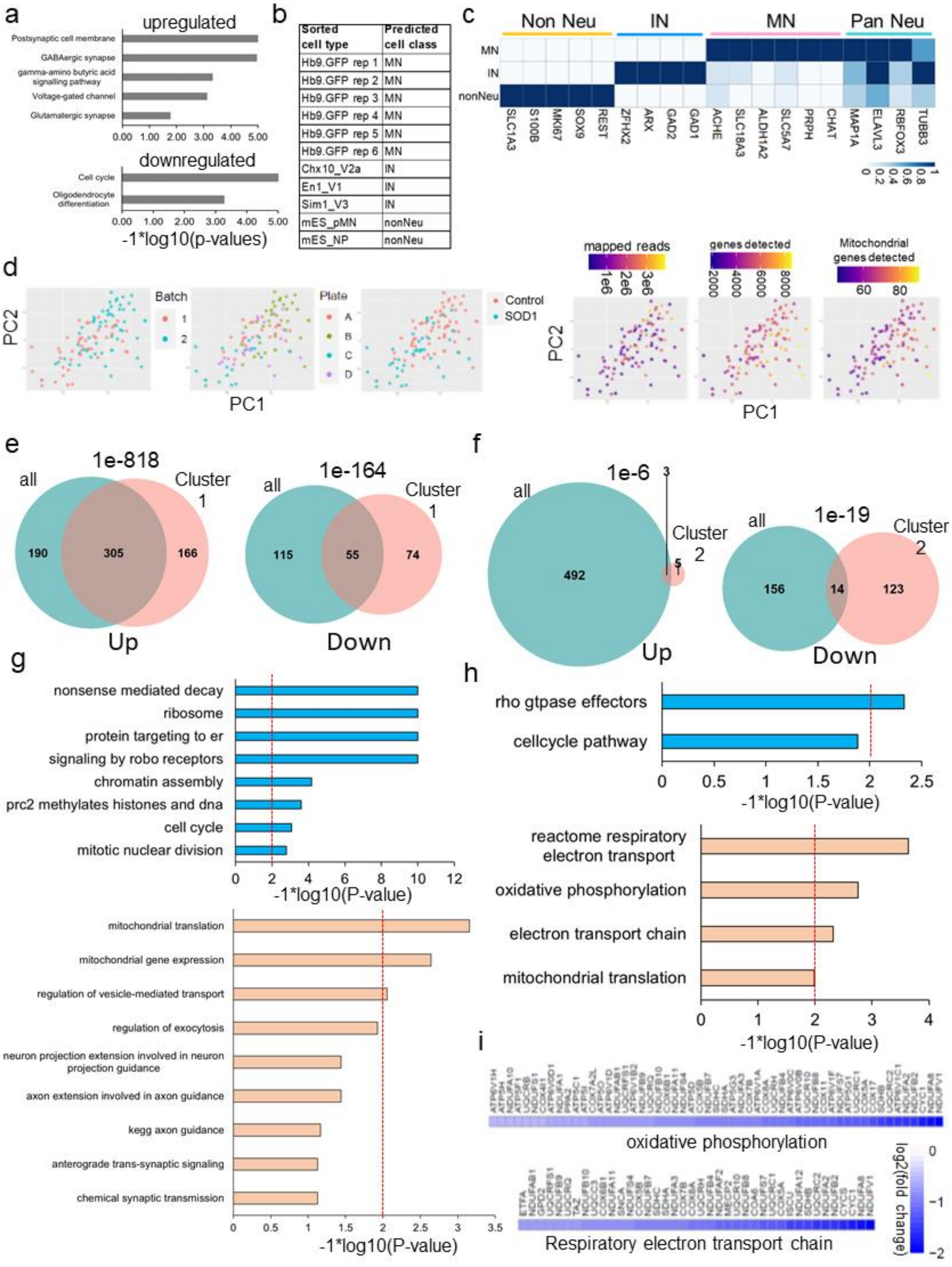

Fig. S2. Classification and differential gene expression of single cell data

a) GO enrichment analysis of neuronal vs glial classifier gene set. b) Validation of the classification process using an independent dataset (GSE75599). The classifier correctly identifies the three key classes: MNs, INs and non-neuronal cells. c) Classification of cells as MNs, INs, and non-neuronal cells based on the external dataset of marker genes. Normalized mean expression of neural markers across the three classes. Pan Neu: pan neuronal markers, Non Neu: non-neuronal markers. d) PCA plots generated using all expressed genes for the MN subset showing distribution of batch, plate, genotype, mapped reads, proportion of mapped reads and number of mitochondrial genes detected. e,f) Overlap between genes up or downregulated in SOD1 ipMNs in cluster 1 (e) or cluster 2 (f) and all ipMNs. Significance was estimated using the hypergeometric distribution. g,h) Pathways identified using gene set enrichment analysis (GSEA) on genes up (blue bars) or down (orange bars) regulated in SOD1 ipMNs in cluster 1 (g) and cluster 2 (h). p-values were adjusted using the Benjamini Hochberg procedure. i) Leading edge analysis reveals the core set of genes driving the observed enrichment scores in the GSEA for the terms “oxidative phosphorylation” and “respiratory electron transport chain”. The identified genes are nuclear-encoded mitochondrial transcripts. Color indicates log2 fold changes of SOD1 ipMNs vs control ipMNs.

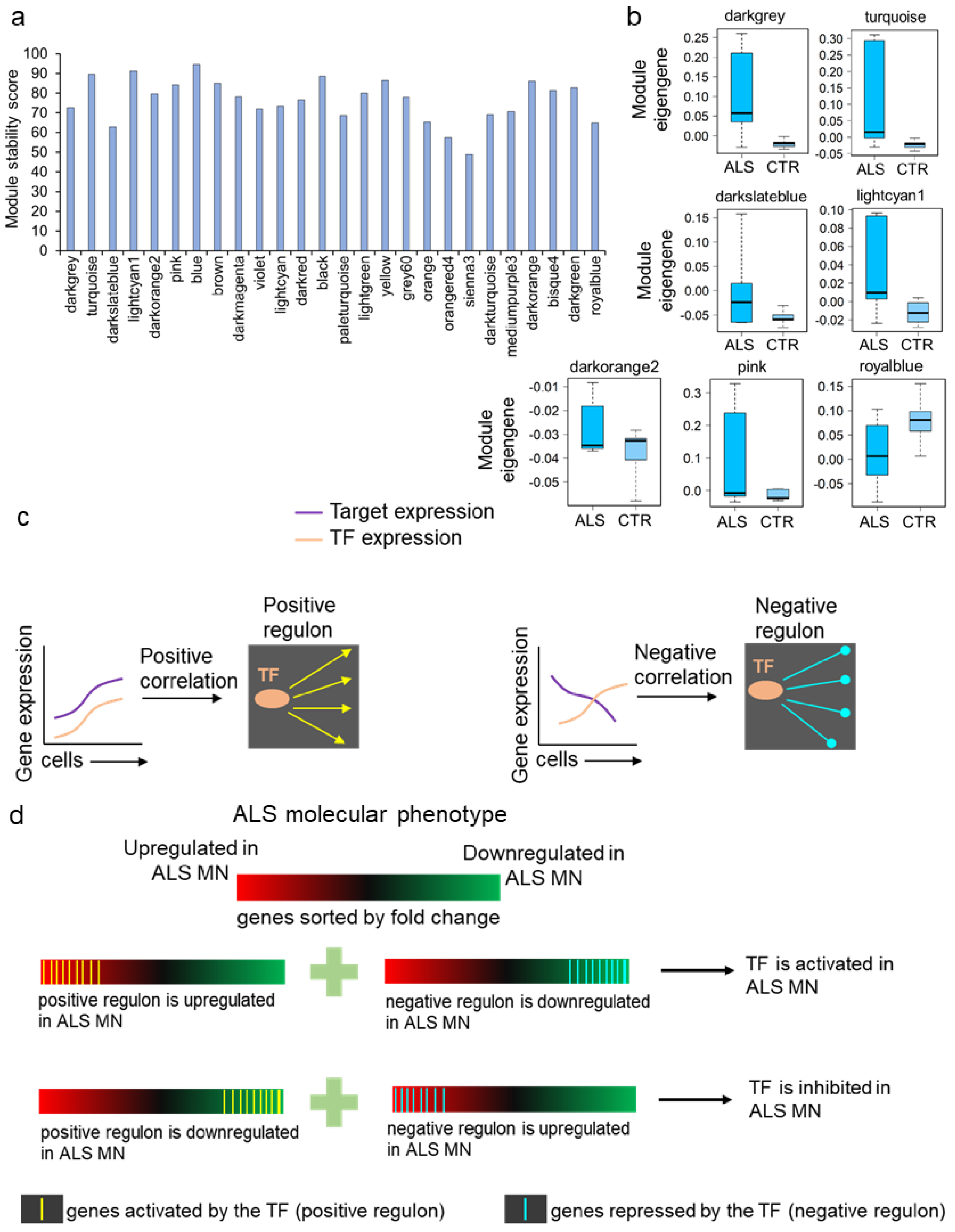

Fig. S3. Assessment of WGCNA identified modules

a) Bar plot showing stability scores for each module. Scores were estimated as described in Supplementary methods. b) Module eigengenes (the first principal component of the

expression of genes assigned to a module) were used to represent the overall expression of the module. Boxplots show the distribution of the module eigengene values between SOD1 and Control (CTR) ipMNs for the modules identified to be significantly associated with SOD1 ipMNs. Positively associated modules: darkgrey, turquoise, darkslateblue, lightcyan1, darkorange2, pink. Negatively associated module: royalblue. c) Generating a transcriptional network using ARACNE. The algorithm estimates pairwise mutual information (MI) scores between TFs and potential target genes, and estimates a significance threshold (p-value) based on a null distribution of scores. MI scores that pass this threshold are deemed valid TF-target pairs. The MI scores do not have a direction i.e. they are always positive. To estimate directionality of regulation, ARACNE estimates correlation between the TF and its target. A positive correlation indicates the TF activates the target while a negative correlation indicates that the TF represses the target. The positive targets of a TF are termed as the positive regulon while the negative targets are deemed the negative regulon. d) Master regulator analysis to identify drivers of SOD1 ipMN dysregulation. Genes differentially expressed in SOD1 ipMNs are sorted from most upregulated to most downregulated. The algorithm then estimates the enrichment of the positive and negative regulons of each TF across the sorted gene expression dataset. For a given TF, if the positive regulon is enriched in the upregulated end of the sorted genelist and the negative regulon is enriched in the downregulated end, the TF is assigned a positive score. If the inverse is true, the TF is assigned a negative score. TFs with significant positive scores are deemed to be activated in SOD1 ipMNs and are most likely to drive the SOD1 ipMN gene expression. On the other hand, TFs with significant negative scores are deemed to be inhibited in SOD1 ipMNs. The significance of a score is estimated using random simulations.

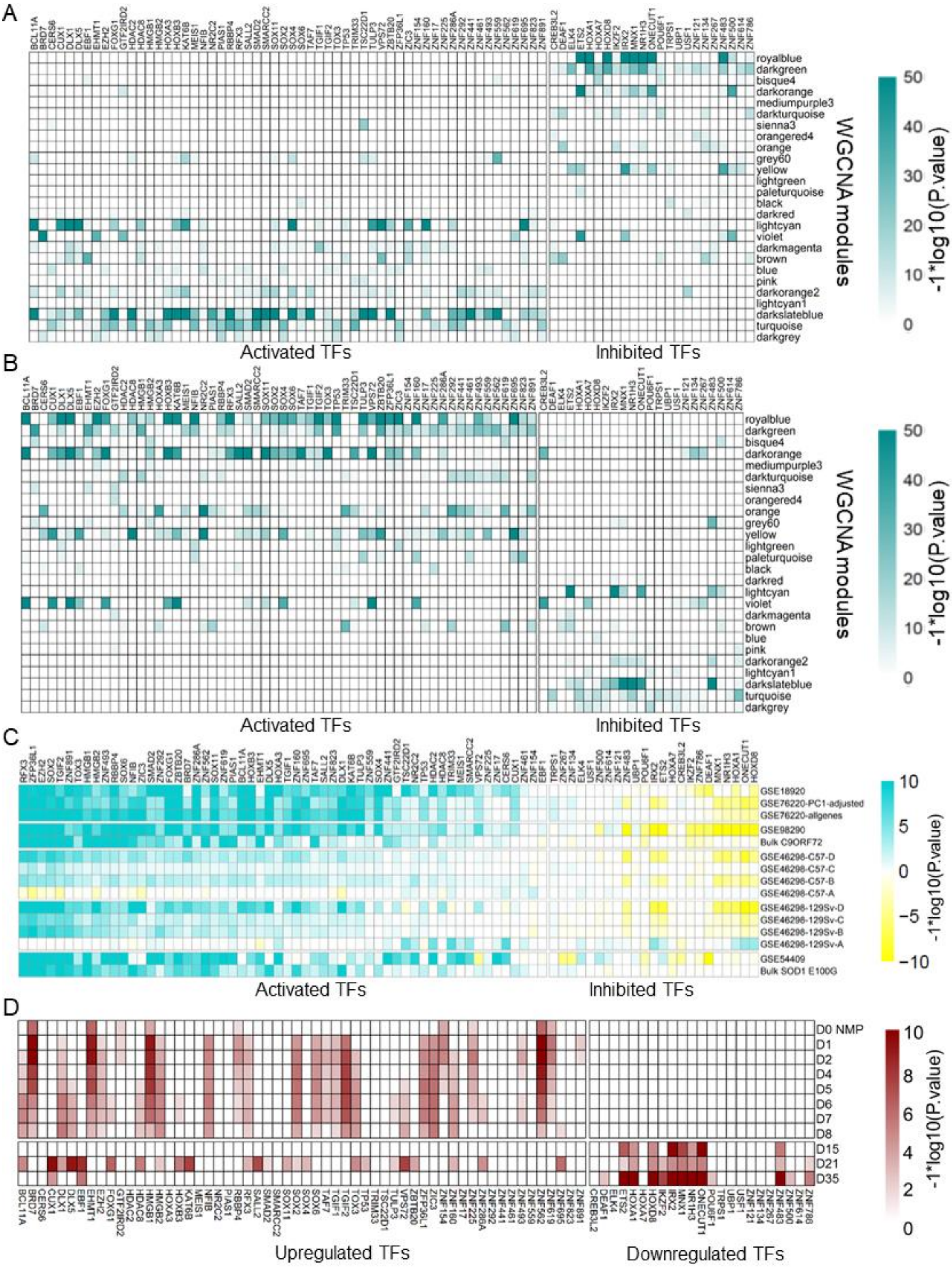

Fig. S4. Overlap analysis of TF regulons

a,b) Overlap between regulons of the 81 TFs identified as master regulators of SOD1 ipMN gene dysregulation and WGCNA modules. (a) analysis using the positive regulons of the TFs. (b) analysis using the negative regulons of the TFs. Significance of the overlap was estimated using a hypergeometric distribution. P-values were adjusted using the Benjamini Hochberg procedure, log transformed and plotted as a heatmap. c) Enrichment of the positive regulons of the 81 master regulator TFs in publicly available ALS datasets. The datasets are as described in Table 1. Positive regulons were used as input genesets for a GSEA performed on each dataset. Log transformed P-values were assigned the same sign as the GSEA enrichment scores and plotted as a heatmap. Green indicates that the regulon was activated while yellow indicates that the regulon was inhibited in the queried dataset of differentially expressed genes. d) Progenitor gene expression programs are reactivated in ALS MN. Enrichment of the positive regulons of the 81 master regulators identified by the MRA. Enrichment was estimated using a one-way GSEA where the positive regulons of each TF were used as genesets. The differentially expressed sorted progenitor and MN genelists were the same as those used in figure 3. Heatmap shows log transformed p-values.

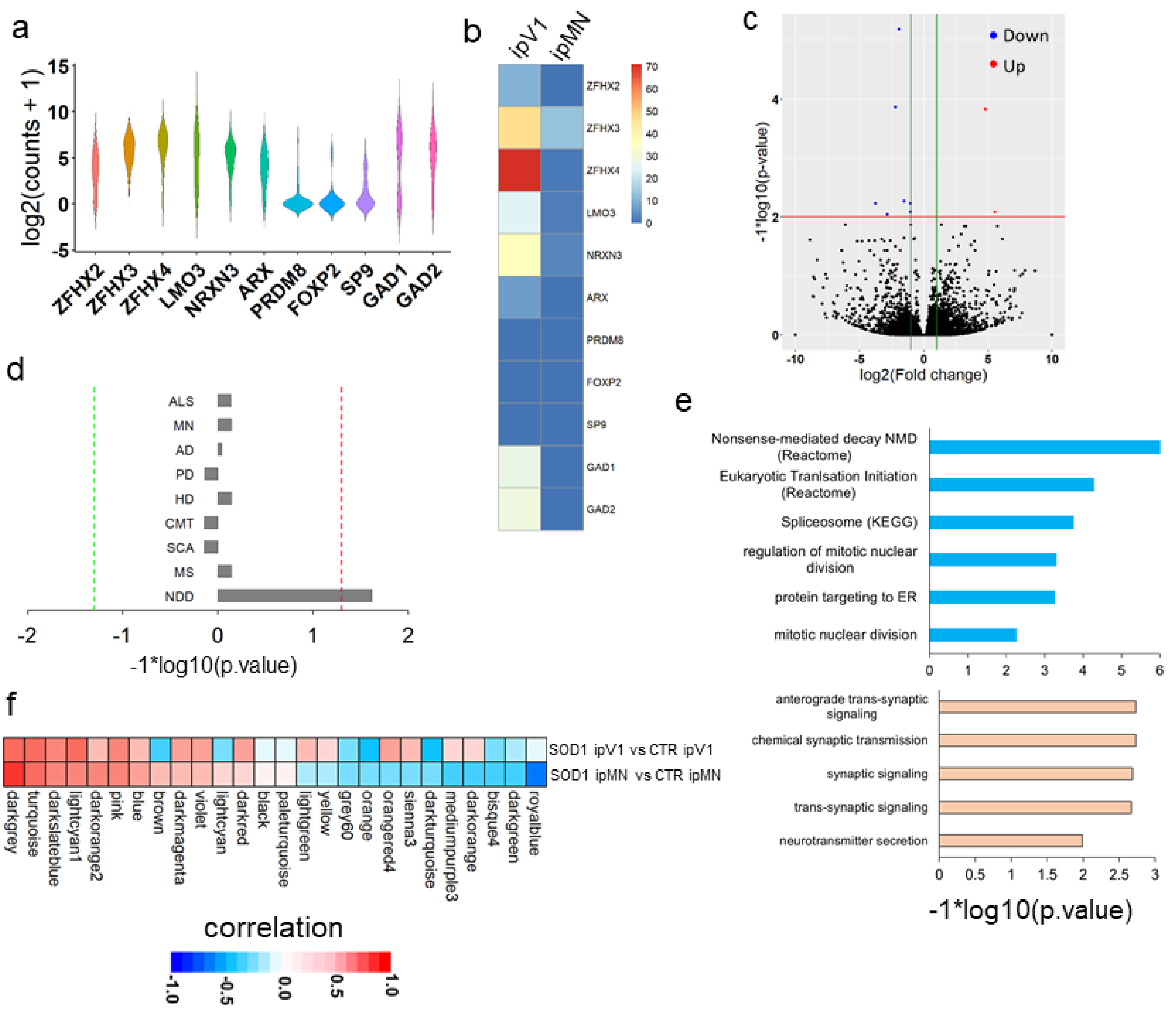

Fig. S5. Differential gene expression analysis of SOD1 V1 INs

a) Violin plot showing log2 transformed expression values of genes known to be expressed in V1 ipINs. b) Normalized mean expression of V1 markers between V1 ipINs and ipMNs. c) Volcano plot of genes differentially expressed in SOD1 V1 ipINs. Each dot represents a gene. Red: upregulated, blue: downregulated, black: unchanged genes. Horizontal red line indicates a p-value of 0.01. Vertical green lines indicate a log2(fold change) = 1. Fold changes >10 or <-10 were set 10 and -10 respectively. All p-values were adjusted using the Benjamini Hochberg procedure. d) Enrichment analysis of likely pathogenic variants associated with different diseases in genes upregulated in SOD1 V1 ipINs. Vertical axis shows terms used to search the ClinVar database to find associated pathogenic variants. ALS: Amyotrophic Lateral Sclerosis, MN: Motor neuron, AD: Alzheimers disease, PD: Parkinsons disease, HD: Huntingtons

disease, CMT: Charcot Marie Tooth, SCA: Spinocerebellar Ataxia, MS: Multiple Sclerosis, NDD: Neurodevelopment disorder. The red and green dashed lines indicate a p-value threshold of 0.05. Values on the right side of the axis indicate enrichment in upregulated genes while values on the left indicate enrichment in downregulated genes. e) Enrichment of GO terms in genes upregulated (blue bars) or downregulated (orange bars) in SOD1 V1 ipINs. The red dashed line indicates a p-value threshold of 0.01. f) Correlation analysis of WGCNA module eigengenes SOD1 V1 ipINs and ipMNs. Red: positive correlation, blue: negative correlation.

Table S1. Primary antibodies used for immunofluorescence. Related figures 1,5.

Target	Source	Catalog number	Dilution
ISL1	Abcam	ab8650	1:500
ISL1	Abcam	ab109517	1:500
MAP2	Abcam	ab11267	1:1000
NF-H	Sigma	N4142	1:1000
Phospho-SMAD2	Cell signalling technology	18338	1:200
KI-67	Cell signalling technology	9449	1:200

Table S2: Primer sequences used for qPCR analysis. Related to figure 5.

Gene	Forward primer	Reverse primer
TGIF1	GGATTGGCTGTATGAGCACCGT	GCCATCCTTTCTCAGCATGTCAG
TGIF2	CTGAGCCTTTCTGGACAGACCA	GGTCTTTGCCATCCTTCCGAAG
HMGA2	GAAGCCACTGGAGAAAAACGGC	GGCAGACTCTTGTGAGGATGTC
VIM	AGGCAAAGCAGGAGTCCACTGA	ATCTGGCGTTCCAGGGACTCAT
MYO10	CACTCTGCCGTATTTCCACAGC	TTTGTGGAGCCAGCCTTGCTTG
MEGF10	TGACTGCTTGCCTGGCTTCACA	GTTACAGGTTCCGTTGTTGGTGC
HES1	GGAAATGACAGTGAAGCACCTCC	GAAGCGGGTCACCTCGTTCATG
ITGA5	GCCGATTCACATCGCTCTCAAC	GTCTTCTCCACAGTCCAGCAAG
TGFBI	GGACATGCTCACTATCAACGGG	CTGTGGACACATCAGACTCTGC
TGFB1	GTGAGGTCCACGGAAACTGT	TGGCTGGTGCAAAGACATAG
ZFP36L1	CAGGATTCTCTCTCGGACCAG	CAGGCGTCTTGAGTTGTCCA
HPRT1	TGCTCGAGATGTGATGAAGG	AATCCAGCAGGTCAGCAAAG
RPL13	CCTGGAGGAGAAGAGGAAAGAGA	TTGAGGACCTCTGTGTATTTGTCAA

Supplementary datafile S1.xls

Genes affected by ambient expression.

Supplementary datafile S2.xls

Marker expression matrix to classify cell types.

Amin ND, Bai G, Klug JR, Bonanomi D, Pankratz MT, Gifford WD, Hinckley CA, Sternfeld MJ, Driscoll SP, Dominguez B et al. 2015. Loss of motoneuron-specific microRNA-218 causes systemic neuromuscular failure. *Science* **350**: 1525-1529.

Angelidis I, Simon LM, Fernandez IE, Strunz M, Mayr CH, Greiffo FR, Tsitsiridis G, Ansari M, Graf E, Strom TM et al. 2019. An atlas of the aging lung mapped by single cell transcriptomics and deep tissue proteomics. *Nat Commun* **10**: 963.

Bhinge A, Namboori SC, Zhang X, VanDongen AMJ, Stanton LW. 2017. Genetic Correction of SOD1 Mutant iPSCs Reveals ERK and JNK Activated AP1 as a Driver of Neurodegeneration in Amyotrophic Lateral Sclerosis. *Stem Cell Reports* **8**: 856-869.

Delile J, Rayon T, Melchionda M, Edwards A, Briscoe J, Sagner A. 2019. Single cell transcriptomics reveals spatial and temporal dynamics of gene expression in the developing mouse spinal cord. *Development* **146**.

Gautier L, Cope L, Bolstad BM, Irizarry RA. 2004. affy--analysis of Affymetrix GeneChip data at the probe level. *Bioinformatics* **20**: 307-315.

Hall CE, Yao Z, Choi M, Tyzack GE, Serio A, Luisier R, Harley J, Preza E, Arber C, Crisp SJ et al. 2017. Progressive Motor Neuron Pathology and the Role of Astrocytes in a Human Stem Cell Model of VCP-Related ALS. *Cell Rep* **19**: 1739-1749.

Harrow J, Frankish A, Gonzalez JM, Tapanari E, Diekhans M, Kokocinski F, Aken BL, Barrell D, Zadissa A, Searle S et al. 2012. GENCODE: the reference human genome annotation for The ENCODE Project. *Genome Res* **22**: 1760-1774.

Huang da W, Sherman BT, Lempicki RA. 2009. Bioinformatics enrichment tools: paths toward the comprehensive functional analysis of large gene lists. *Nucleic Acids Res* **37**: 1-13.

Kiskinis E, Sandoe J, Williams LA, Boulting GL, Moccia R, Wainger BJ, Han S, Peng T, Thams S, Mikkilineni S et al. 2014. Pathways disrupted in human ALS motor neurons identified through genetic correction of mutant SOD1. *Cell Stem Cell* **14**: 781-795.

Krach F, Batra R, Wheeler EC, Vu AQ, Wang R, Hutt K, Rabin SJ, Baughn MW, Libby RT, Diaz-Garcia S et al. 2018. Transcriptome-pathology correlation identifies interplay between TDP-43 and the expression of its kinase CK1E in sporadic ALS. *Acta Neuropathol* **136**: 405-423.

Luisier R, Tyzack GE, Hall CE, Mitchell JS, Devine H, Taha DM, Malik B, Meyer I, Greensmith L, Newcombe J et al. 2018. Intron retention and nuclear loss of SFPQ are molecular hallmarks of ALS. *Nat Commun* **9**: 2010.

Nardo G, Iennaco R, Fusi N, Heath PR, Marino M, Trolese MC, Ferraiuolo L, Lawrence N, Shaw PJ, Bendotti C. 2013. Transcriptomic indices of fast and slow disease progression in two mouse models of amyotrophic lateral sclerosis. *Brain* **136**: 3305-3332.

Rabin SJ, Kim JM, Baughn M, Libby RT, Kim YJ, Fan Y, Libby RT, La Spada A, Stone B, Ravits J. 2010. Sporadic ALS has compartment-specific aberrant exon splicing and altered cell-matrix adhesion biology. *Hum Mol Genet* **19**: 313-328.

Rayon T, Stamataki D, Perez-Carrasco R, Garcia-Perez L, Barrington C, Melchionda M, Exelby K, Tybulewicz V, Fisher WMC, Briscoe J. 2019. Species-specific developmental timing is associated with global differences in protein stability in mouse and human. *BioRxiv* doi:https://doi.org/10.1101/2019.12.29.889543.

Ritchie ME, Phipson B, Wu D, Hu Y, Law CW, Shi W, Smyth GK. 2015. limma powers differential expression analyses for RNA-sequencing and microarray studies. *Nucleic Acids Res* **43**: e47.

Selvaraj BT, Livesey MR, Zhao C, Gregory JM, James OT, Cleary EM, Chouhan AK, Gane AB, Perkins EM, Dando O et al. 2018. C9ORF72 repeat expansion causes vulnerability of motor neurons to Ca(2+)-permeable AMPA receptor-mediated excitotoxicity. *Nat Commun* **9**: 347.

Shannon CP, Chen V, Takhar M, Hollander Z, Balshaw R, McManus BM, Tebbutt SJ, Sin DD, Ng RT. 2016. SABRE: a method for assessing the stability of gene modules in complex tissues and subject populations. *BMC Bioinformatics* **17**: 460.

Wagner F, Yan Y, Yanai I. 2018. K-nearest neighbor smoothing for high-throughput single-cell RNA-Seq data. *bioRxiv* doi:https://doi.org/10.1101/217737.
Zhang Y, Sloan SA, Clarke LE, Caneda C, Plaza CA, Blumenthal PD, Vogel H, Steinberg GK, Edwards MS, Li G et al. 2016. Purification and Characterization of Progenitor and Mature Human Astrocytes Reveals Transcriptional and Functional Differences with Mouse. *Neuron* **89**: 37-53.
