# Supplementary material for: Single-cell transcriptomics identifies master regulators of neurodegeneration in SOD1 ALS iPSC-derived motor neurons
Source: Stem Cell Reports. 2021 Nov 11;16(12):3020–35. doi: 10.1016/j.stemcr.2021.10.010 (PMC8693652; doi:10.1016/j.stemcr.2021.10.010)
Supplement: Document S2. Article plus supplemental — information [file mmc4.pdf]

Stem Cell Reports
Article
OPEN ACCESS

Single-cell transcriptomics identifies master regulators of neurodegeneration in SOD1 ALS iPSC-derived motor neurons

Seema C. Namboori,[1,4] Patricia Thomas,[2] Ryan Ames,[3] Sophie Hawkins,[1,4] Lawrence O. Garrett,[1] Craig R.G. Willis,[5] Alessandro Rosa,[6,7] Lawrence W. Stanton,[8] and Akshay Bhinge[1,4,*]
[1]Living Systems Institute, University of Exeter, Exeter EX4 4QD, UK
[2]Institute of Metabolism and Systems Research, Birmingham Medical School, University of Birmingham, Birmingham B15 2TT, UK
[3]Biosciences, University of Exeter, Exeter EX4 4QD, UK
[4]College of Medicine and Health, University of Exeter, Exeter EX1 2LU, UK
[5]Department of Sport and Health Sciences, College of Life and Environmental Sciences, University of Exeter, Exeter EX1 2LU, UK
[6]Department of Biology and Biotechnologies "Charles Darwin", Sapienza University of Rome, P.le A. Moro 5, 00185 Rome, Italy
[7]Center for Life Nano- & Neuro-Science, Fondazione Istituto Italiano di Tecnologia (IIT), Viale Regina Elena 291, 00161 Rome, Italy
[8]Qatar Biomedical Research Institute, Hamad Bin Khalifa University, Doha, Qatar
*Correspondence: a.bhinge@exeter.ac.uk
https://doi.org/10.1016/j.stemcr.2021.10.010

SUMMARY

Amyotrophic lateral sclerosis (ALS) is a fatal neurodegenerative condition characterized by the loss of motor neurons. We utilized single-cell transcriptomics to uncover dysfunctional pathways in degenerating motor neurons differentiated from SOD1 E100G ALS patient-derived induced pluripotent stem cells (iPSCs) and respective isogenic controls. Differential gene expression and network analysis identified activation of developmental pathways and core transcriptional factors driving the ALS motor neuron gene dysregulation. Specifically, we identified activation of SMAD2, a downstream mediator of the transforming growth factor β (TGF-β) signaling pathway as a key driver of SOD1 iPSC-derived motor neuron degeneration. Importantly, our analysis indicates that activation of TGFβ signaling may be a common mechanism shared between SOD1, FUS, C9ORF72, VCP, and sporadic ALS motor neurons. Our results demonstrate the utility of single-cell transcriptomics in mapping disease-relevant gene regulatory networks driving neurodegeneration in ALS motor neurons. We find that ALS-associated mutant SOD1 targets transcriptional networks that perturb motor neuron homeostasis.

INTRODUCTION

Amyotrophic lateral sclerosis (ALS) is an age-onset, fatal, incurable neurodegenerative disorder that affects motor neurons (MNs) in the brain and spinal cord. Patients display progressive paralysis and eventually die due to respiratory failure, commonly within 3–5 years of diagnosis (Brown and Al-Chalabi 2017). Despite extensive research, the causes underlying the observed degeneration are incompletely understood. Understanding the molecular drivers of neurodegeneration in ALS can potentially lead to the development of life-saving therapies. Approximately 20% of ALS cases are familial with mutations identified in genes spanning diverse cellular functions, including *SOD1* (Hardiman et al., 2017).

Patient-derived induced pluripotent stem cells (iPSCs) bear the disease-causing mutations in a physiologically relevant background and provide a powerful model to study ALS. These iPSCs can be differentiated into MNs to model key aspects of the disease, such as neuron survival, morphometric and electrophysiological defects, and protein/RNA aggregation foci *in vitro* (Fujimori et al., 2018; Lopez-Gonzalez et al., 2016; Selvaraj et al., 2018).

Molecular characterization of these neurons using "omics" tools has uncovered important insights into disease pathophysiology (De Santis et al., 2017; Krach et al., 2018). However, application of genomic tools such as RNA sequencing (RNA-seq) to ALS neurons in bulk has serious drawbacks. Current differentiation protocols generate spinal MNs at efficiencies ranging from 50% to 80% depending upon the iPSC line used. The differentiated neurons are usually a mix of MNs, spinal interneurons (INs), or glial cells. To dissect cell-type-specific differences, we performed single-cell transcriptomic analysis of degenerating ALS SOD1 iPSC-derived neurons. Our single-cell data enabled us to build a disease-relevant transcriptional network that was used to identify key transcription factors driving the ALS-associated gene dysregulation.

RESULTS

Single-cell RNA-seq analysis of iPSC-derived SOD1 and control neurons

We differentiated iPSCs derived from patients bearing the SOD1 E100G mutation, as well as the CRISPR edited isogenic control (SOD1 E100E) into MNs as described previously (Figure 1A) (Bhinge et al., 2017). Day 30 neurons expressed the MN markers ISL1 and NF-H as well as the pan-neuronal marker MAP2 (Figure 1B). Using our protocol, both SOD1 and control iPSCs could be reproducibly differentiated into MNs (termed ipMNs) at similar efficiencies (~75% ISL1+ cells) (Figure 1B). Between days 30 and 44, SOD1 ipMNs displayed a 40% loss in survival

 © 2021 The Authors.
This is an open access article under the CC BY license (http://creativecommons.org/licenses/by/4.0/).

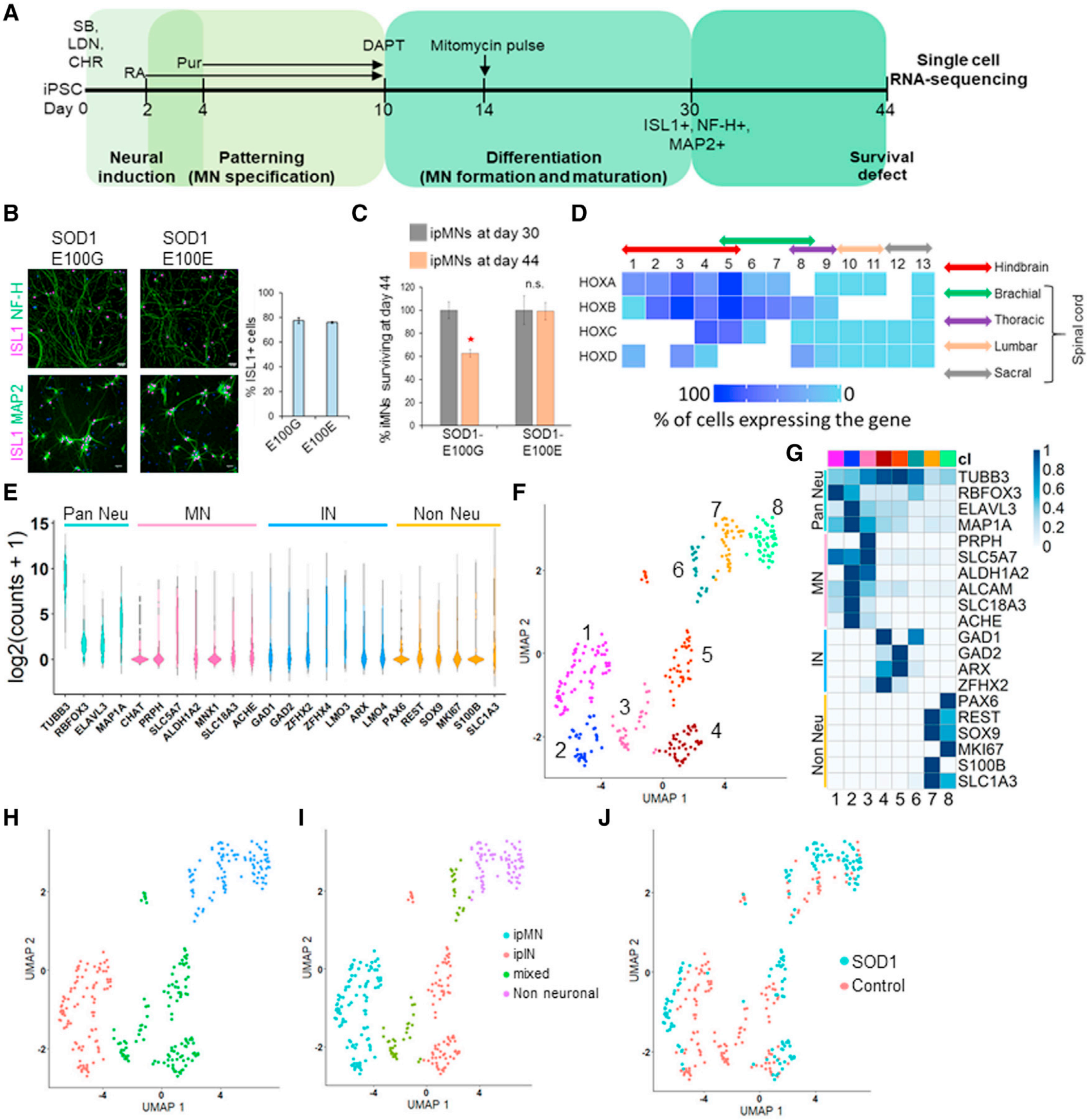

Figure 1. Single-cell RNA-seq of iPSC-derived neurons

(A) Differentiation protocol used to derive MNs from human iPSCs. Numbers on the horizontal line indicate days. SB, SB431542; LDN, LDN193189; CHR, CHIR99021; RA, retinoic acid; Pur, purmorphamine.

(B) ipMNs at day 30 stain positive for ISL1, NF-H, and MAP2. Scale bar indicates 50 μm.

(C) MNs (ISL1+) were counted at d30 and d44 of the differentiation protocol. D44 MN counts were normalized to d30 counts. SOD1 E100G indicates the MNs derived from mutant SOD1 iPSC. SOD1 E100E indicates the isogenic corrected control MNs. Error bars shown are SEM, n = 3 independent biological replicates. *$p < 0.01$; n.s., not significant.

(D) Heatmap displaying percentage of cells expressing specific *HOX* genes. White space indicates that the corresponding *HOX* paralog is not expressed in humans. Colored solid arrows indicate the *HOX* code for specific spinal segments along the rostro-caudal axis.

(E) Violin plots displaying distribution of expression levels of displayed markers across all cells.

(F) UMAP plot showing clustering of single cells.

(G) Normalized mean expression of neural markers across all eight clusters (cl). Clusters have been coded by numbers (at the bottom) and by color (at the top). These correspond to the numbers and colors shown in (F).

(legend continued on next page)

compared with the isogenic control ipMNs (Figure 1C). To gain deeper insights into the mechanisms driving neurodegeneration, we performed single-cell RNA-seq on the SOD1 and isogenic control ipMNs at day 44 of our differentiation protocol. We captured a total of 332 single cells across two independent differentiations, which included 165 cells from the SOD1 and 167 cells from the isogenic cultures (Supplemental experimental procedures). After removing low-quality cells (Supplemental experimental procedures, Figure S1), we retained 163 cells for SOD1 and 160 cells for the control (323 cells total). Our single-cell transcriptomes for the SOD1 and control sets were similar in quality on a genome-wide level (Supplemental experimental procedures, Figure S1).

In vivo, spinal MNs at different rostro-caudal levels of the spinal cord are demarcated by specific combinations of *HOX* gene expression (known as the *HOX* code) (Philippidou and Dasen 2013). To ascertain the rostro-caudal address of our *in vitro* differentiated neurons, we estimated the percentage of cells expressing each of the 39 *HOX* genes (Figure 1D). Most cells expressed *HOXA5* and *HOXB5*, with few cells expressing *HOXB8* and *HOXD8* and none expressing *HOX* genes from paralog groups 9 and higher (Figure 1D). This indicated that our cells were largely restricted to the hindbrain or brachial spinal cord identity. Next, we assessed expression of markers for motor neurons (*MNX1*, *CHAT*, *PRPH*, *SLC5A7*, *ALDH1A2*, *ACHE*, *VAChT*, or *SLC18A3*), interneurons (*GAD1*, *GAD2*, *ZFHX2*, *ZFHX4*, *LMO3*, *LMO4*, *ARX*), and non-neuronal cells (*PAX6*, *REST*, *MKI67*, *S100B*, *SOX9*, *SLC1A3*) (Figure 1E). Our data indicated that iPSC-derived neuronal cultures display wide variation in expression across individual cells that is averaged in bulk analysis. To resolve this heterogeneity and enable differential expression between relevant classes of neurons, we sought to classify cells into specific neural lineages.

Classification of single cells into neural subtypes

We first identified genes that could be used to classify cells into relevant cell types (neurons versus glia and MNs versus INs) (Supplemental experimental procedures, Figures S2A–S2C). This set of 1,060 genes, termed the classifier gene set, was used to cluster cells into distinct neural subtypes. We performed unsupervised clustering of all single cells (healthy and SOD1) using our classifier gene set into eight clusters using uniform manifold approximation and projection (UMAP) as part of the Monocle3 package (Becht et al., 2018; Cao et al., 2019) (Figure 1F). Analysis of median expression of known MN, IN, and non-neuronal marker genes confirmed successful clustering of cells by subtypes; i.e., MNs (clusters 1, 2, and 3), INs (clusters 4, 5, and 6), and non-neuronal glial progenitors (clusters 7 and 8) (Figure 1G). To evaluate the possibility of mixed clusters (i.e., cells that display expression patterns of multiple cell types), we performed partition analysis, which groups clusters together into "super-clusters." Partition analysis grouped cluster 6 (IN cluster) with the non-neuronal clusters 7 and 8, while the MN cluster 3 was grouped together with the IN clusters 4 and 5 (Figure 1H). Hence, cells in clusters 3 and 6 were termed mixed and removed from further analysis (Figure 1I). Our clustering approach identified 61 MNs and 41 INs in the healthy dataset, while 38 MNs and 49 INs were identified in the SOD1 ALS dataset (Figure 1J).

Differential expression analysis of SOD1 and control neurons

We compared gene expression in our SOD1 ipMNs with the isogenic control ipMNs using DESeq with parameters recommended for single-cell RNA-seq (see section, "experimental procedures"). Principal component analysis (PCA) on the MN subset indicated the absence of any batch effects in terms of differentiation or bias in mapped reads, number of detected genes, and mitochondrial genes (Figure S2D). Differential expression analysis identified 495 upregulated genes and 170 downregulated genes in SOD1 ipMNs at a p value <0.01 (adjusted for multiple hypothesis correction) and fold change >2 (Figures 2A, 2B, and 2E). On the other hand, analysis of SOD1 and control ipINs revealed far fewer genes dysregulated in SOD1 ipINs compared with SOD1 ipMNs at the same threshold (63 genes upregulated and 46 genes downregulated) (Figures 2C, 2D, and 2E). There was minimal, although significant, overlap in the differentially expressed genes between ipMNs and ipINs, with 30 genes shared in the upregulated set and four genes shared in the downregulated set (Figure 2F). The differentially expressed genes in ipINs had a similar distribution for the fold changes, but most genes did not pass the p value threshold (Figure 2C). This could be due to multiple interneuron subtypes present in the population. Hence, we first decided to focus on MNs for further analysis.

(H) Partition analysis shows the MN cluster 3 associating closely with IN clusters 4 and 5, while IN cluster 6 associates with non-neuronal clusters 7and 8.
(I) UMAP plot showing classification of single cells into MNs, Ins, and non-neuronal cells based on marker expression.
(J) UMAP plot showing distribution of the identified cell types among the ALS SOD1 E100G (SOD1) and isogenic SOD1 E100E control samples.
See also Figures S1 and S2.

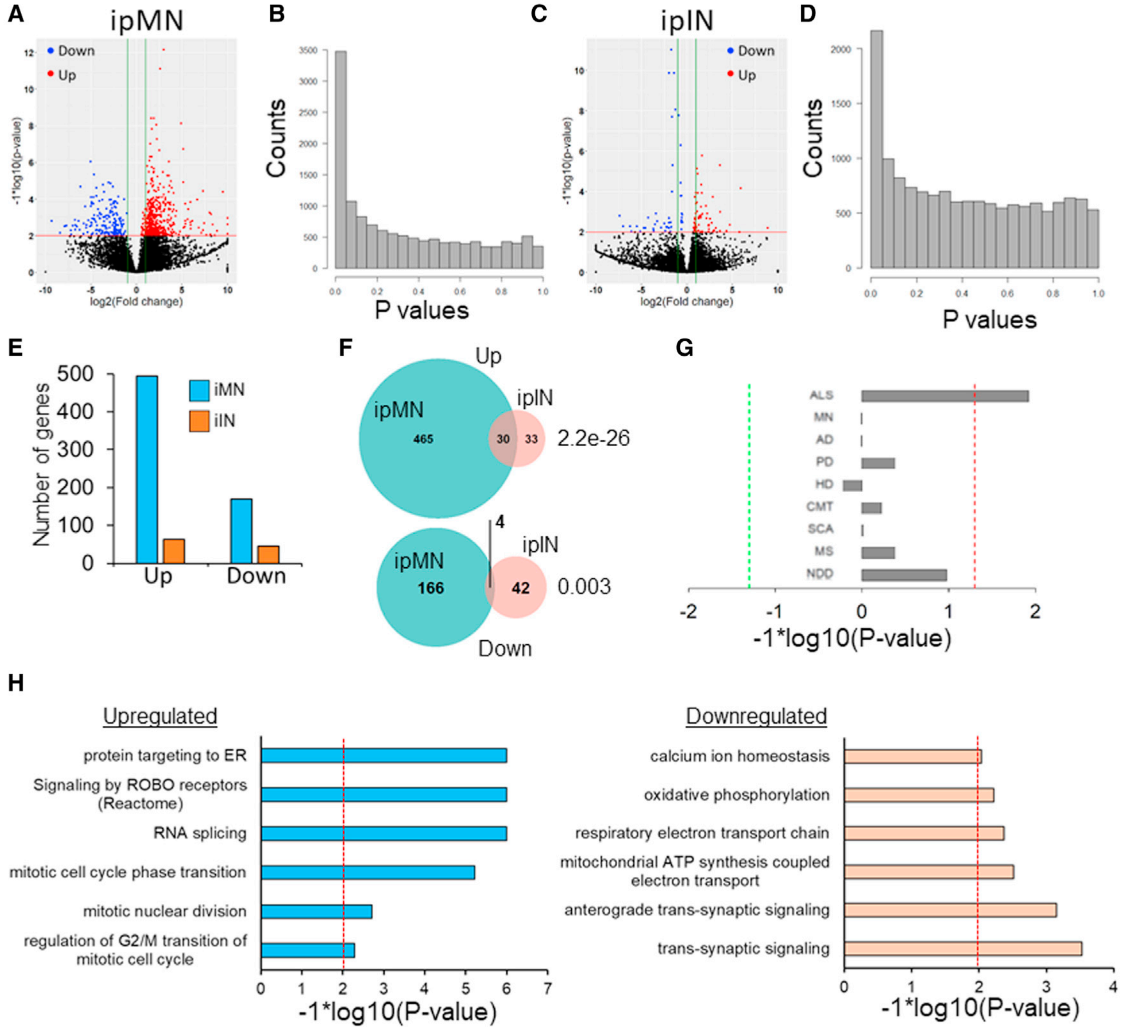

Figure 2. Differential expression analysis of SOD1 ipMNs and ipINs

(A and C) Volcano plots of differentially expressed genes (A) for ipMNs and (C) ipINs. Each dot represents a gene. Red, upregulated genes; blue, downregulated genes; black, unchanged genes. Horizontal red line represents p value = 0.01. Vertical green lines represent an absolute log2 fold change of 1. Fold changes >10 or <−10 were set 10 and −10 respectively.

(B and D) p value histograms of the differentially expressed genes for (B) ipMNs and (D) ipINs.

(E) Number of genes up- or downregulated in SOD1 ipMNs (blue) compared with SOD1 ipINs (orange).

(F) Overlap between genes up- or downregulated between SOD1 ipMNs and ipINs. Significance was estimated using the hypergeometric distribution.

(G) Enrichment analysis of likely pathogenic variants associated with different diseases in genes upregulated in SOD1 ipMNs. Vertical axis shows terms used to search the ClinVar database to find associated pathogenic variants. PD, Parkinson disease; HD, Huntington disease; CMT, Charcot-Marie-Tooth; SCA, spinocerebellar ataxia; MS, multiple sclerosis; NDD, neurodevelopment disorder. The red and green dashed lines indicate a p value threshold of 0.05. Values on the right side indicate enrichment in upregulated genes, while values on the left indicate enrichment in downregulated genes.

(H) Pathways identified using GSEA in genes upregulated (left) or downregulated (right) in SOD1 ipMNs. p values were adjusted using the Benjamini-Hochberg procedure.

See also Figure S2.

We observed that pathogenic variants associated with ALS in the ClinVar (Landrum et al., 2020) database were significantly enriched, specifically in genes upregulated in the SOD1 ipMNs (Figure 2G). Gene set enrichment analysis (GSEA) identified several pathways dysregulated in SOD1 ipMNs. The downregulated pathways included terms related to synaptic function ("trans-synaptic signalling" and "anterograde trans-synaptic signalling") and

mitochondrial function ("respiratory electron transport" and "oxidative phosphorylation") (Figure 2H). Additionally, calcium homeostasis was also downregulated in SOD1 ipMNs. Pathways enriched in the upregulated genes included the mitotic cell cycle, protein targeting to the ER, and RNA splicing (Figure 2H). The observed changes in gene expression were not driven by differential cluster membership of SOD1 and control ipMNs (Figures S2E–S2H, Supplemental experimental procedures). Interestingly, we observed that downregulation of the terms "oxidative phosphorylation" and "respiratory electron transport" were driven by nuclear-encoded as opposed to mitochondrial-encoded genes (Figure S2I).

Co-regulated gene modules dysregulated in SOD1 compared with healthy neurons

To gain a systems-level understanding of the observed transcriptional changes, we performed weighted gene coexpression network analysis (WGCNA) (Langfelder and Horvath 2008). WGCNA identifies sets of genes that are highly correlated (called gene modules) and links these modules with specific phenotypic traits associated with each sample. However, since single-cell data are highly sparse, performing correlation analysis on the read counts might lead to spurious associations or false-negatives. Hence, we implemented a k-nearest neighbors (k-NN) algorithm to smooth the read counts (Wagner et al., 2018) (Supplemental experimental procedures). Further, we only retained genes that were expressed in $\geq$20 cells. The smoothened normalized count data across 189 neurons and 14,054 genes were used to construct modules using topological overlap. WGCNA identified 26 gene modules comprising 81–1,444 genes (median = 449 genes) (Figure 3A). Using a bootstrapping approach (Supplemental experimental procedures), we confirmed that 19 out of the 26 modules had a stability score >70 (maximum 100), while only two modules scored <60 (Figure S3A). We used the module eigengene (the first principal component for all genes in a given module) as a representative expression of that module in a cell. By estimating the Pearson correlations of each module eigengene with cell type or genotype, we identified modules that were positively or negatively associated with ipMNs, ipINs, or disease status.

Six modules (darkgrey, turquoise, darkslateblue, lightcyan1, darkorange2, pink) were positively correlated while one module (royal blue) was negatively correlated to SOD1 ipMNs (adjusted p value <0.01 and absolute correlation >0.4) (Figures 3B and S3B). All seven modules associated with SOD1 ipMNs had stability scores >60 (Figure S3A). Gene ontology (GO) enrichment analysis of these modules revealed association of each module with specific functional categories (Figure 3C). Genes assigned to the royalblue module were significantly enriched for synaptic function and signaling, axon structure, autophagy, and respiratory electron transport. The turquoise module was enriched in genes associated with mitotic cell cycle, TP53 activation, and chromatin remodeling. The darkslateblue module was enriched in terms related to RNA processing, including translation, splicing, and decay. The pink module was enriched in the terms "coagulation" and "Signalling by Wnt". The darkgrey module showed enrichment of terms related to catalytic activity that included chromatin remodeling enzymes. Interestingly, the royalblue module associated significantly with healthy ipMNs compared with healthy ipINs (Figure 3B), indicating that genes involved in synaptic function and respiration are highly expressed in ipMNs compared with ipINs, in accordance with the high metabolic demands on MNs.

Network analysis using WGCNA has previously revealed disruption of age-related modules and pathways in sporadic ALS MNs (Ho et al., 2016). For example, the expression of genes involved in translation decreased with age but was upregulated in sporadic ALS MNs. We observed that the module darkslateblue, which positively correlated with SOD1 ipMNs, was enriched in genes with a role in translation. This led us to question whether SOD1 ipMNs displayed aberrant reactivation of developmental pathways. To test this hypothesis, we obtained gene expression data over the time course of differentiation from neuromesodermal progenitors (NMPs; D0) to MNs (D15) (Rayon et al., 2019) as well as immature (D21) and mature (D35) neurons (Hall et al., 2017; Luisier et al., 2018). D0 to D8 genelists displayed progenitor-enriched genes at the top, while D15, D21, and D35 genelists displayed neuron-enriched genes at the top (Supplemental experimental procedures). We used GSEA to quantify the enrichment of the WGCNA modules in each sorted genelist (Figure 3D). As hypothesized, module darkslateblue was significantly enriched in the progenitor genelists D0 to D8, indicating that genes assigned to this module are highly active earlier in motor neuron development. Modules turquoise and lightcyan1, which were activated in SOD1 ipMNs, were also enriched at earlier developmental time points. Remarkably, genes in module royalblue that were downregulated in SOD1 ipMNs were enriched in the D15, D21, and D35 neuron-enriched genelists. This indicated that SOD1 ipMNs display activation of genes highly enriched in progenitors compared with mature neurons. Accordingly, genes upregulated in SOD1 ipMNs were significantly enriched in genelists D0 to D8, while downregulated genes were enriched in the neuronal genelists D15, D21, D35 (Figure 3E).

Next, we assessed module enrichment in genes upregulated in published ALS datasets (Figure 3F; Table1). The turquoise module was enriched in the iPSC-derived SOD1 A4V MNs, SOD1 mouse model (129Sv), C9ORF72 and VCP

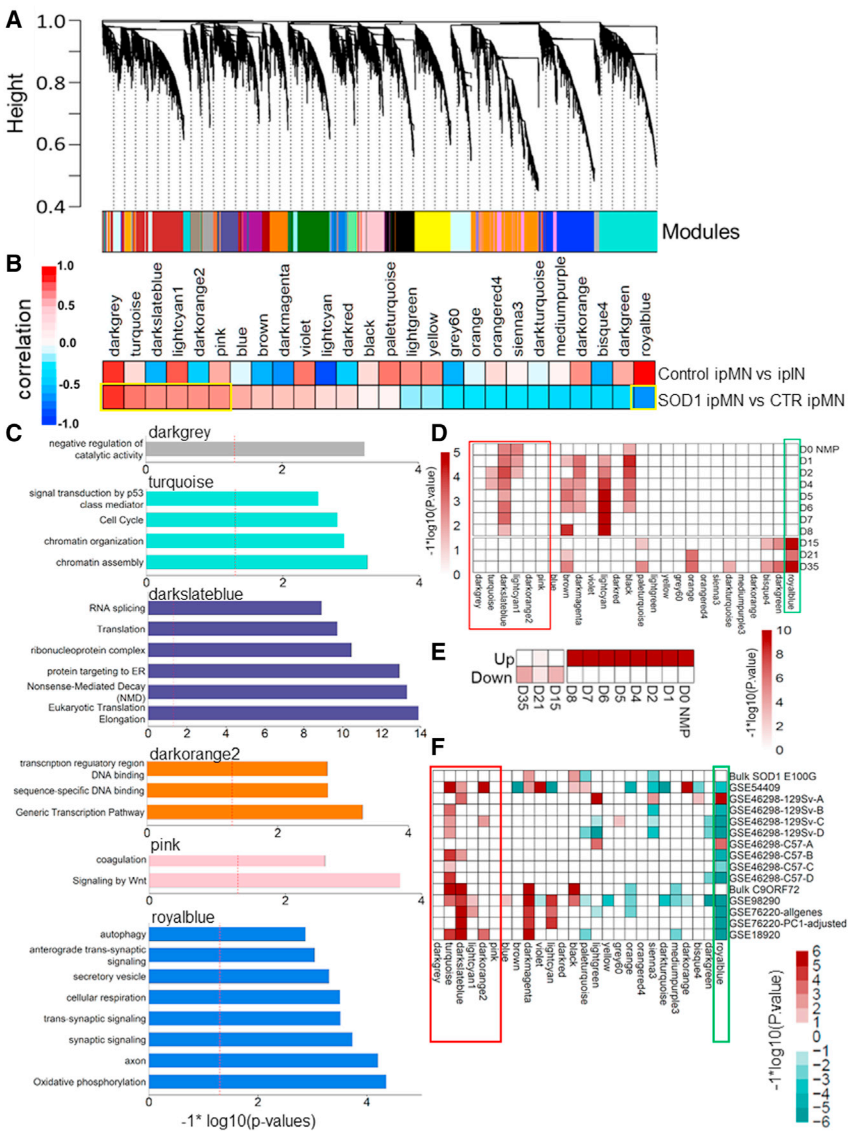

Figure 3. WGCNA

(A) WGCNA identified 26 modules marked by specific colors across the neuronal dataset. Height indicates the dissimilarity between genes, which was based on topological overlap.

(B) Module eigengenes for each module were compared between two groups of cells: i.e., SOD1 ipMN versus control (CTR) ipMN or control ipMN versus ipIN. For a comparison of group A versus group B, red (positive correlation) indicates that the module eigengenes were higher in group A compared with group B, while blue (negative correlation) indicates the reverse. Correlations with an adjusted p value <0.01 were considered significant (outlined in yellow).

(C) GO enrichment analysis of modules significantly correlated (positive or negative) with SOD1 ALS ipMNs. p values shown were adjusted using the Benjamini-Hochberg procedure. Module lightcyan1 did not yield any significant GO terms and is not shown. The red dashed line indicates a p value threshold of 0.05.

(D) Enrichment of WGCNA modules in genes upregulated in neural progenitors compared with neurons. D0 indicates NMPs. D1 to D8 indicate intermediate time points as the NMP differentiated into MN, while D15, D21, and D35 indicate genes enriched in MNs. The red rectangle highlights modules activated in SOD1 ipMNs, while the green rectangle highlights the single module downregulated in SOD1 ipMNs. Enrichments were estimated using a one-way GSEA. Heatmap shows log-transformed p values.

(E) GSEA performed was similar to (D), where genes upregulated (Up) or downregulated (Down) in SOD1 ipMNs were used as gene sets for the GSEA.

(F) Enrichment of WGCNA modules in publicly available ALS datasets (Table 1). The x axis shows the modules. The red rectangle highlights modules activated in SOD1 ipMNs, while the green rectangle highlights the single module downregulated in SOD1 ipMNs. Log-transformed p values were assigned the same sign as the GSEA enrichment scores and plotted as a heatmap. Red indicates positive enrichment, while green indicates negative enrichment of a module in the queried dataset.

See also Figure S3.

iPSC-derived MNs, and the GEO: GSE18920 sporadic ALS MN datasets. The darkslateblue module was also enriched in the iPSC-derived SOD1 A4V, C9ORF72 and VCP iPSC-derived MNs, and at the onset stage in both SOD1 mouse models (129Sv and C57). Remarkably, both turquoise and darkslateblue modules were upregulated in at least one of the sporadic ALS datasets. This enrichment was observed even after removal of genes involved in wound healing for the GEO: GSE76220 dataset (Figure 3F). The royalblue module was significantly downregulated in SOD1 and VCP iPSC-derived MNs, mouse SOD1 MNs, and sporadic ALS MNs. Surprisingly, the bulk SOD1 E100G dataset did not show enrichment of the turquoise or darkslateblue modules. This dataset is derived from bulk RNA-seq analysis without purifying MNs from the iPSC-derived neuronal cultures, which could have reduced the sensitivity of detection.

Master regulator analysis

We wanted to identify transcription factors (TFs) that were main drivers (termed master regulators) of the molecular changes in SOD1 ipMNs. Since each cell can be considered

Table 1. ALS gene expression datasets

Name	Description	Reference
Bulk SOD1 E100G	SOD1 E100G iPSC-derived MNs analyzed in bulk	Bhinge et al. (2017)
GEO: GSE54409	SOD1 A4V iPSC-derived MNs purified via flow sorting	Kiskinis et al. (2014)
GEO: GSE46298-129Sv	MN laser-capture micro-dissected from SOD1 G93A mouse model fast-progressing strain 129Sv. A, pre-symptomatic; B, onset; C, symptomatic; D, end stage	Nardo et al. (2013)
GEO: GSE46298-C57	MN laser-capture micro-dissected from SOD1 G93A mouse model slow-progressing strain C57. A, pre-symptomatic; B, onset; C, symptomatic; D, end stage	Nardo et al. (2013)
Bulk C9ORF72	C9ORF72 and isogenic iPSC-derived MNs analyzed in bulk	Selvaraj et al. (2018)
GEO: GSE98290	VCP and healthy iPSC-derived MNs analyzed in bulk	Hall et al. (2017)
GEO: GSE76220	MN laser-capture micro-dissected from sporadic ALS spinal lumbar tissue	Krach et al. (2018)
GEO: GSE76220-PC1	GEO: GSE76220 data were filtered for genes involved in wound healing	Krach et al. (2018)
GEO: GSE18920	MN laser-capture micro-dissected from sporadic ALS spinal lumbar tissue	Rabin et al. (2010)

an independent sample, we used our single-cell data to build a context-relevant transcriptional network to identify master regulators (Ikiz et al., 2015). We deployed the ARACNE algorithm on the k-NN smoothened count data to infer transcriptional networks (Basso et al., 2005; Fletcher et al., 2013). After pre-filtering genes that displayed minimal change in expression across the dataset, downstream targets were identified for 1,137 TFs among 12,550 genes expressed in neurons.

We deployed our network analysis to identify master regulators of SOD1 ipMN dysfunction (Figure S3C and S3D). Genes differentially expressed between SOD1 and isogenic control ipMNs were used to define a molecular phenotype of the disease. Master regulators were identified based on whether there was a statistically significant overlap between the positive and negative regulon of a TF and the SOD1 molecular phenotype. For a given TF, if the positive regulon was upregulated and the negative regulon was downregulated in SOD1 ipMNs, the TF was deemed to be activated. If the inverse was true, the TF was deemed to be inhibited. The master regulator analysis (MRA) identified ~200 TFs at an FDR <0.01. To further filter the candidate regulators, we checked for concordance between the expression change of a TF and its regulon, and filtered out non-concordant TFs (i.e., where the direction of change of the regulon expression and the TF expression is not the same). We also filtered out TFs that were not differentially expressed in the SOD1 ipMNs compared with control. This identified a core set of 81 TFs (58 activated and 23 inhibited) that satisfied the following criteria: (1) the TFs were differentially expressed in SOD1 ipMNs compared with control, (2) the regulons of these TFs showed significant association (positive or negative) with the SOD1 gene expression signature, (3) the TF and its regulon expression was concordant (Figure 4A). The identified master regulators included *TP53*, *HMGB2*, *TGIF1*, and *ZFP36L1* as potential drivers of SOD1. All of these TFs have been implicated as disease drivers in SOD1 ALS, validating our approach (Bhinge et al., 2017; Ikiz et al., 2015).

To further investigate these regulators, we extracted subnetworks of the activated and inhibited TFs and clustered them on the basis of their mutual information scores (Figures 4B and 4C). The inhibited TFs formed two clusters where the second cluster (highlighted in red) showed higher co-regulation compared with the first cluster, indicating that these TFs functioned in similar pathways (Figure 4B). This cluster included two *HOX* genes, *HOXA1* and *HOXD8*. *HOX* genes have important roles in defining MN identity during development (Philippidou and Dasen 2013). Although *HOX* genes have been found to be expressed in adult human and mouse MNs (Nichterwitz et al., 2016), their function in MNs post specification is

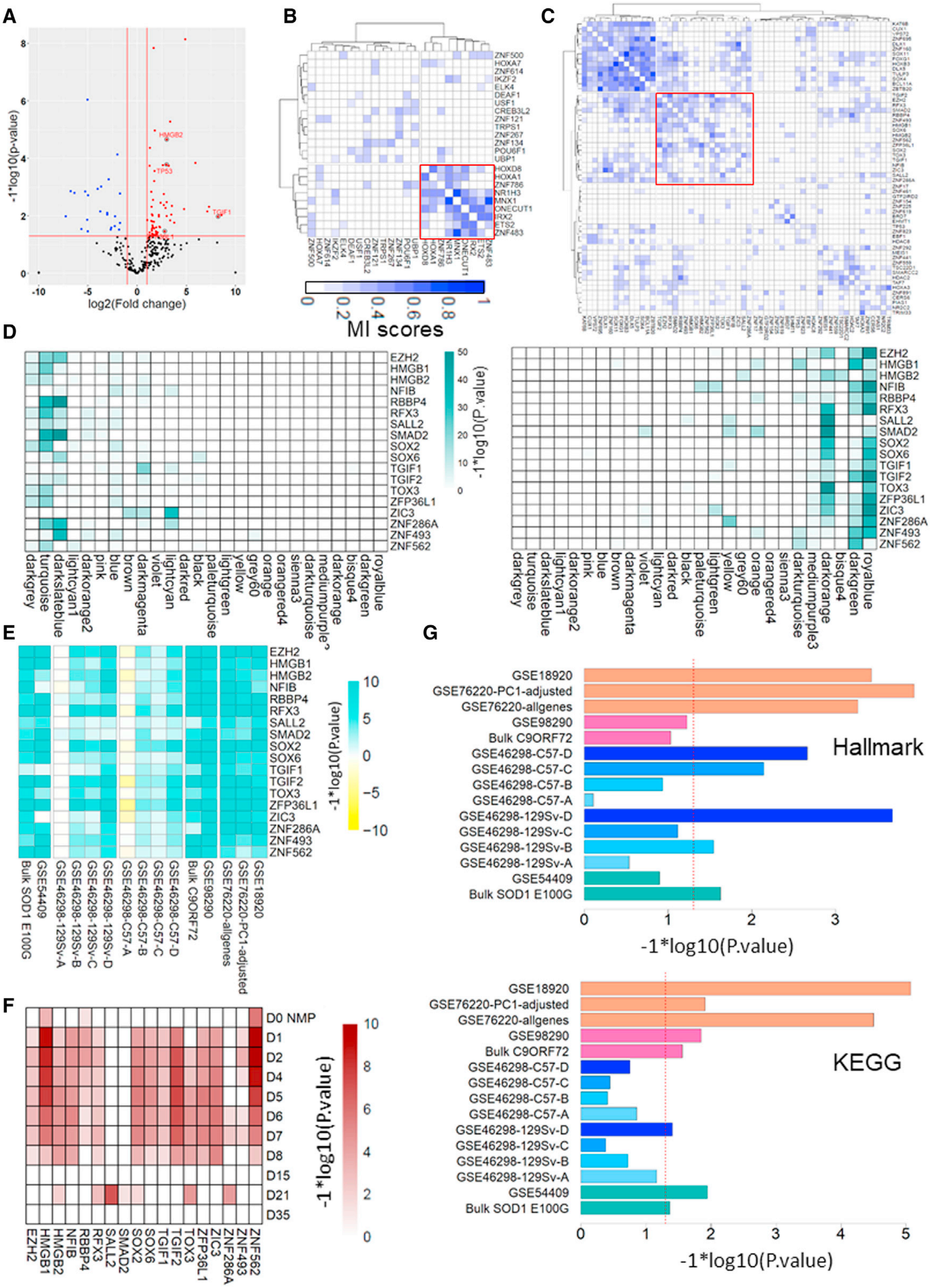

(legend on next page)

unclear. This cluster also included the TFs *MNX1* and *ONECUT1*, which are known to be involved in MN homeostasis. This suggested that master regulators of MN homeostasis may be downregulated in degenerating SOD1 ipMNs. However, we noted that *MNX1* was lowly expressed in our dataset (read counts were ≤3 in 91 out of the 99 MNs), which can exaggerate the fold changes identified by DESeq2. Nevertheless, our data indicate that *MNX1* should be used with caution as a marker to identify MNs while performing survival analyses.

For the activated TFs, clustering analysis broadly identified four clusters (Figure 4C). The second cluster (highlighted in red) included the TF *SMAD2*, a key mediator of the transforming growth factor β (TGFβ) signaling pathway. This cluster also included other TFs known to be involved in TGFβ signaling (*TGIF1*, *TGIF2*), their downstream targets (*ZFP36L1*, *SOX2*), as well as *EZH2*, a member of the PRC2 complex that works synergistically with the TGFβ pathway (Martin-Mateos et al., 2019; Massague 2012; Pastar et al., 2010; Weina et al., 2016). TGFβ signaling has previously been observed to be upregulated in spinal astrocytes and muscle of transgenic SOD1 mouse models of ALS (Endo et al., 2015; Si et al., 2015). Hence, we decided to focus on the 18 TFs identified in cluster 2.

To investigate a link between the identified TFs and WGCNA modules, we calculated the overlap between the positive regulons and modules using a hypergeometric test. We found several TFs whose targets showed significant overlap with the seven modules (darkgrey, turquoise, darkslateblue, lightcyan1, darkorange2, pink, and royalblue) associated with SOD1 ipMNs (Figures S4A and S4B). Out of the 18 TFs in cluster 2 in Figure 4C, the positive regulons of 15 TFs showed a significant overlap, with the top three modules (darkgrey, turquoise, and darkslateblue) deemed to be upregulated in SOD1 ipMNs (Figure 4D left panel). The *SMAD2* positive regulon displayed significant overlap with both the turquoise and darkslateblue modules. The negative regulons of 16 out the 18 TFs, including *EZH2*, *TGIF1*, *TGIF2*, *SOX2*, and *ZFP36L1*, significantly overlapped with the royalblue module, which was downregulated in SOD1 ipMNs (Figure 4D right panel). This indicated that the identified master regulators were driving specific gene expression programs in SOD1 ipMNs.

Next, we assessed whether identified master regulators were dysregulated in other ALS datasets using GSEA (Figure S4C; Table 1). The positive regulons of most of our activated master regulator TFs (56 out of 58) were activated in at least one ALS dataset (Figure S4C). Regulons of TFs related to MN homeostasis and identity such as *MNX1*, *ONECUT1*, *HOXA1*, and *HOXD8* were downregulated in sporadic ALS MNs and in the end-stage SOD1 G93A 129Sv MNs but not in MNs derived from ALS SOD1 iPSC (Figure S4C). This suggested that downregulation of MN homeostatic regulators could be a terminal event in dying neurons.

TFs associated with TGFβ signaling (*SMAD2*, *TGIF1*, *TGIF2*, *ZFP36L1*, *EZH2*, *HMGB1*, and *SOX2*) were activated in familial ALS iPSC-derived MNs (SOD1, VCP, C9ORF72), SOD1 mouse models, and sporadic ALS MNs (Figure 4E). Additionally, *SMAD2* was activated in all ALS models, including mouse SOD1 G93A MNs at the onset stage (Figure 4E). Finally, we observed that the positive regulons of

Figure 4. MRA of SOD1 disease signature

(A) Volcano map showing differential expression of concordant TFs identified by the MRA. Each dot is a TF. Red indicates upregulation. Blue indicates downregulation. Black indicates no significant change of expression. TFs previously associated with SOD1 MN degeneration are highlighted in gray circles (*TGIF1*, *HMGB2*, *TP53*, *ZFP36L1*).

(B) Clustering of master regulators inhibited in SOD1 ipMNs based on their mutual information (MI) scores. Higher MI score indicates co-regulation between TFs and is highlighted blue. Red square outlines a cluster of TFs that are highly co-regulated.

(C) Clustering of master regulators activated in SOD1 ipMNs based on their MI scores. Higher MI scores are indicated in blue. Red square outlines a cluster of TFs associated with TGFβ signaling (*SMAD2*, *TGIF1*, *TGIF2*, *ZFP36L1*, *EZH2*, *HMGB1*, and *SOX2*).

(D) Overlap analysis between regulons and WGCNA modules. (Left) Positive regulons of the 18 TFs highlighted in red in (C) were compared with gene modules identified by WGCNA. Log-transformed p values were plotted as a heatmap. (Right) Same analysis performed using the negative regulons of the displayed TFs.

(E) Heatmap showing the enrichment of the positive regulons of the 18 TFs highlighted in (C) in publicly available ALS datasets (Table 1). Positive regulons of the TFs were used as gene sets for a GSEA performed on each dataset. Log-transformed p values were assigned the same sign as the GSEA enrichment scores and plotted as a heatmap. Green, regulon was activated; yellow, regulon was downregulated in the queried dataset.

(F) Enrichment of the positive regulons of the 18 TFs highlighted in (C) in genes differentially activated in progenitors and neurons. Enrichment was estimated using a one-way GSEA where the positive regulons of each TF were used as gene sets. Heatmap shows log-transformed p values.

(G) Enrichment of the TGFβ signaling pathway in publicly available ALS MN datasets estimated by performing GSEA. TGFβ pathway datasets were derived from the MSigDB hallmark and KEGG databases, and were used as the input gene set. The red dashed line indicates a p value threshold of 0.05.

See also Figure S3 and S4.

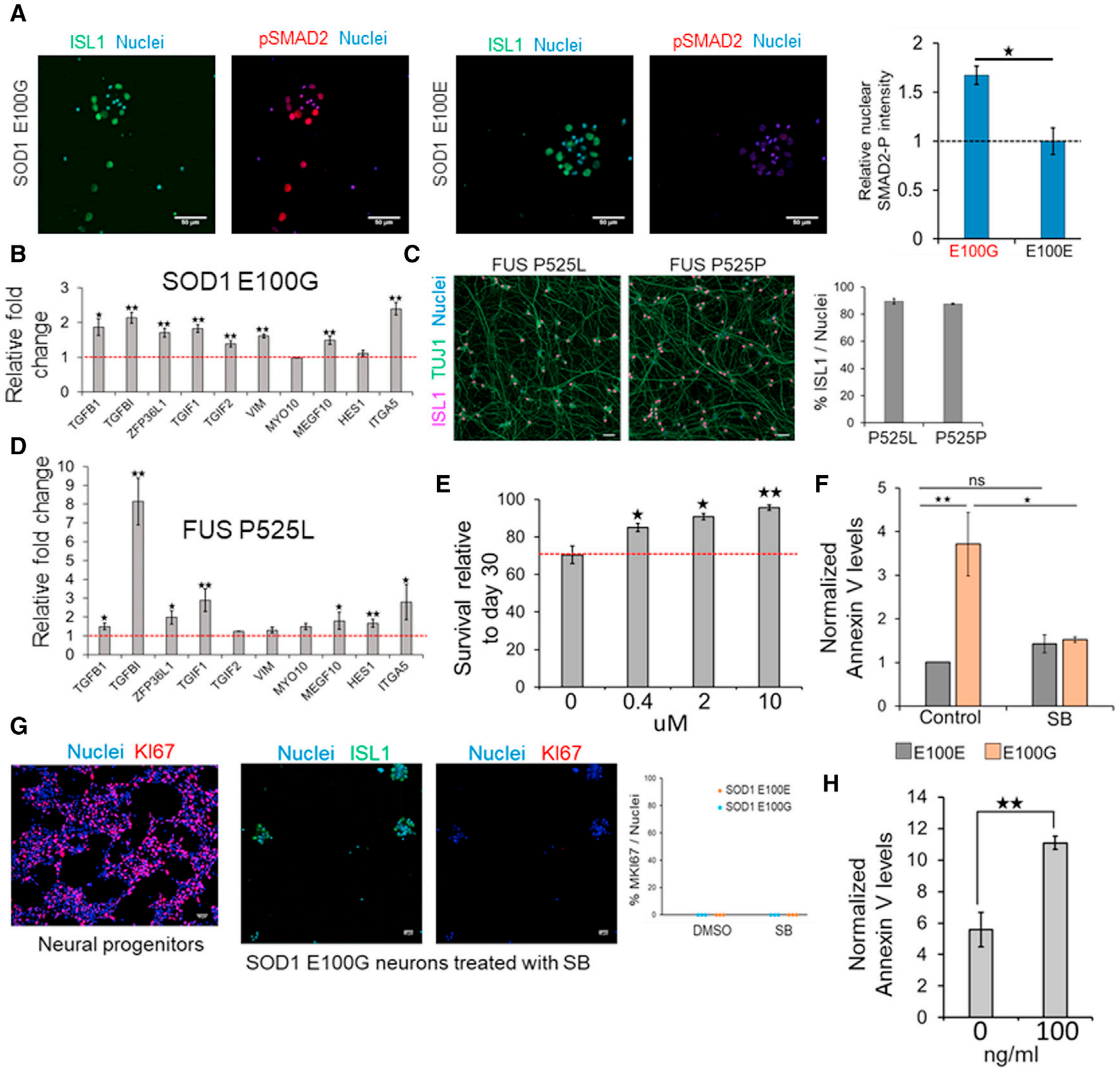

Figure 5. TGFβ signaling is a key driver of SOD1 MN degeneration

(A) Immunofluorescence analysis of phosphorylated SMAD2 (p-SMAD2) and ISL1 in SOD1 ALS (E100G) and isogenic control (E100E) neuronal cultures. Nuclei were stained with Hoechst 33,342. p-SMAD2 intensities in ISL1+ nuclei were quantified across three independent differentiations for the SOD1 and control cultures. Median intensity values per nuclei were estimated across at least 100 nuclei per replicate and averaged. Values obtained for each replicate were normalized by the average values in the control neurons.

(B) Quantitative RT-PCR analysis of TGFβ targets in SOD1 E100G ipMNs relative to isogenic controls. p values were estimated using one-tailed Student's t test.

(C) MN differentiated from FUS P525L and isogenic control iPSC. D30 ipMNs were immunostained for ISL1 and TUJ1. Nuclei were stained with Hoechst 33342. FUS P525P indicates MNs differentiated from the isogenic corrected iPSCs.

(D) Quantitative RT-PCR analysis of TGFβ targets in FUS P525L ipMNs relative to isogenic controls. p values were estimated using one-tailed Student's t test.

(E) Quantitation of nuclei in SOD1 neuronal cultures after treatment with SB431542 at the indicated concentrations. Number of cells at day 40 were compared relative to day 30.

(F) Relative annexin V levels in day 40 SOD1 and isogenic control neuronal cultures after treatment with SB431542 10 μM. Annexin V levels were normalized to those obtained in the DMSO-only control.

(G) (Left) D8 progenitors were immunostained with an antibody against the proliferation marker KI67. Strong nuclear signal seen in most cells. (Middle) Representative image of KI67 immunostaining in day 40 SOD1 iPSC-derived neuronal cultures treated with SB431542. Nuclei were stained using Hoechst 33342. (Right) Quantification of KI67+ cells in the SOD1 or control neuronal cultures treated with SB431542 or DMSO at day 40. No KI67+ cells could be detected.

(legend continued on next page)

several master regulators, including TFs activated by the TGFβ pathway (*TGIF1*, *TGIF2*, *ZFP36L1*, *SOX2*), were highly enriched in progenitors (Figures 4F and S4D). This indicated that transcriptional mediators of the TGFβ pathway may be responsible, at least in part, for reactivation of these developmental programs.

Overall, these observations led us to hypothesize that the TGFβ pathway is activated in ALS MNs. We tested this hypothesize by analyzing whether genes involved in TGFβ signaling are upregulated in ALS MNs. We used the TGFβ signaling datasets from the hallmark collection included in the Molecular Signatures Database (MSigDB) (Liberzon et al., 2015) as well as the Kyoto Encyclopedia of Genes and Genomes (KEGG) database (Kanehisa and Goto 2000). Our analysis confirmed that the TGFβ pathway is indeed activated in SOD1 G93A mouse, SOD1 E100G ALS, SOD1 A4V ALS, C9ORF72, VCP, and sporadic ALS MNs (Figure 4G).

TGFβ activation causes phosphorylation and translocation of SMAD2 from the nucleus to the cytoplasm. Immunostaining assays confirmed that SOD1 ipMNs displayed higher levels of nuclear phosphorylated SMAD2 at day 30 relative to control ipMNs (Figure 5A). Further, we confirmed upregulation of TGFβ downstream target genes in the SOD1 ipMNs (Figure 5B), as well as in MNs differentiated from FUS P525L iPSCs compared with their respective isogenic controls (Lenzi et al., 2015) (Figures 5C and 5D). Next, to ascertain whether TGFβ activation contributes to neurodegeneration, we treated SOD1 ipMNs with the TGFβ inhibitor SB431542 at varying concentrations from day 30 until day 40. Treatment with SB431542 significantly enhanced cell survival in a concentration-dependent manner (Figure 5E). Additionally, inhibition of TGFβ signaling decreased apoptotic levels in the SOD1 neural cultures, while isogenic cultures were unaffected (Figure 5F). The increase in cell survival was not due to proliferation of mitotic cells as evidenced by the absence of any nuclear KI67 (MKI67) positive cells in the treated and control cultures (Figure 5G). Conversely, treatment of SOD1 neural cultures with TGFβ from day 30 to day 40 resulted in enhanced apoptosis (Figure 5H). In summary, our results demonstrate that activation of TGFβ signaling leads to degeneration of SOD1 ipMNs and TGFβ activation is a shared event between familial and sporadic ALS MNs.

Analysis of SOD1 V1 ipINs

We investigated whether mutant SOD1 affects human spinal ipINs similar to ipMNs, as shown recently in ALS mouse models (Liu et al., 2020). We classified the 90 ipINs identified in our single-cell data into 49 V1 (26 SOD1 and 23 healthy), nine V2a (one SOD1 and eight healthy), and 21 V2b (16 SOD1 and five healthy) ipINs. The remaining 11 neurons could not be classified into a specific subtype. The V2a and V2b ipIN numbers were skewed toward the healthy and *SOD1* genotypes, respectively. This is probably due to under-sampling of the IN population while collecting single cells. Hence, we focused on the V1 population for further analysis. Spinal V1 INs are a diverse group of neurons that show highly variable gene expression patterns. The V1 neurons identified in our dataset displayed expression of expected V1 markers (Sweeney et al., 2018) (Figures S5A and S5B). Differential expression analysis identified only two genes as upregulated and seven genes to be downregulated in the SOD1 V1 population (Figure S5C). GSEA did not reveal any enrichment of pathogenic variants associated with ALS listed in the ClinVar database (Figure S5D). Surprisingly, genes associated with neurodevelopmental disorders were enriched in genes upregulated in the SOD1 V1 ipINs (Figure S5D). GSEA identified GO terms related to ER stress, mitotic cell cycle, RNA splicing, and translation to be upregulated in SOD1 V1 ipINs, similar to those found in SOD1 ipMNs but with lower enrichment scores (Figure S5E). On the other hand, genes involved in synaptic signaling were downregulated, although to a lower extent than observed in SOD1 ipMNs (Figure S5E). However, terms related to oxidative phosphorylation or respiratory electron transport were not observed to be downregulated in SOD1 V1 ipINs, even when the p value threshold was relaxed to 0.1. Out of the six modules found to correlate positively with SOD1 ipMNs, five (darkgrey, turquoise, darkslateblue, lightcyan1, and pink) were also identified to correlate positively with SOD1 V1 ipINs (Figure S5F). However, the royalblue module (enriched in genes involved in synaptic signaling and respiration) was not deemed to be perturbed in SOD1 V1 ipINs (Figure S5F), indicating that genes involved in oxidative phosphorylation are less affected in SOD1 V1 ipINs compared with ipMNs.

DISCUSSION

ALS patient-derived iPSCs have provided unprecedented access to human diseased MNs, enabling researchers to follow the course of degeneration in a dish. Our ipMNs have a developmental age corresponding to 75–90 days

(H) Relative annexin V levels in day 40 SOD1 neuronal cultures after treatment with TGFβ 100 ng/mL. Annexin V levels were normalized to those obtained in the PBS-only control.
$^{**}p < 0.01$, $^{*}p < 0.05$, N = 3 independent differentiations performed for all experiments. Error bars indicate SEM. p values were estimated using two-tailed Student's t test unless stated otherwise. All scale bars represent 50 μm.

post conception, similar to previous observations (Ho et al., 2016; Steg et al., 2021). However, iPSC-derived neurons display disease-related phenotypes and dysregulated molecular pathways induced by the underlying mutations (Dafinca et al., 2020; Fujimori et al., 2018; Hall et al., 2017; Mehta et al., 2021; Selvaraj et al., 2018). The advent of single-cell genomics has allowed the analysis of individual neurons in mixed cultures (Wang et al., 2017). We have applied this technology to analyze RNA expression in individual neurons derived from SOD1 patient-derived iPSCs and the corresponding isogenic controls. The total number of cells captured in our study is less than typically seen in droplet-based assays. However, we were able to sequence each cell to a greater depth (1.5 million reads per cell). This contrasts with droplet-based studies that result in just 100,000 reads per cell (Ho et al., 2020). As a result, our data identified almost thrice the number of genes per cell than are typically seen in droplet-based experiments. This allowed sensitive identification of differentially expressed genes and pathways. We observed a higher proportion of reads mapping to mitochondrial genes compared with those seen in droplet-based studies, possibly due to our larger depth of sequencing, which would tend to amplify reads from the most abundant transcripts preferentially. Given their high energy demands, MNs can be expected to have higher numbers of mitochondria, thereby contributing to the larger number of reads mapping to mitochondrial transcripts. It is also likely that our SOD1 ipMNs display mitochondrial degeneration at day 44, potentially releasing mitochondrial transcripts into the cell lysate. Mitochondrial degeneration has been observed in mouse MNs expressing mutant SOD1 (Kong and Xu 1998). However, we did not observe an increase in mitochondrial reads in the SOD1 dataset compared with the control.

Defects in synaptic activity and axonal structure have been observed before the onset of neurodegeneration in ALS models (Fischer et al., 2004; Garone et al., 2021; Selvaraj et al., 2018). Synaptic collapse can be a downstream effect of either impaired delivery or production of synaptic proteins and mRNAs. Impaired delivery can occur secondary to inefficient axonal transport (Bilsland et al., 2010). On the other hand, our data reveal inhibition of the synaptic genes at the transcriptional level, possibly due to dysregulation of master regulator TFs in SOD1 ipMNs. Specifically, our analysis implicates SMAD2-mediated TGFβ signaling as a key driver of SOD1 MN dysfunction.

The phenotypic effect of TGFβ activation on neurons seems to be context dependent. For example, inhibiting TGFβ in mouse cortical neurons expressing amyloid-β resulted in neurite degeneration (Tesseur et al., 2006). On the other hand, stimulating TGFβ in cultured mouse hippocampal neurons led to defects in neuronal morphology (Nakashima et al., 2018). This suggests that optimal levels of TGFβ are required to maintain neuronal homeostasis. Our data confirmed that TGFβ activation causes death in SOD1 ALS ipMNs. We observed TGFβ activation in MNs differentiated from iPSCs carrying ALS-associated mutations in FUS (P525L), C9ORF72, and VCP as well as MNs micro-dissected from sporadic ALS patients. This indicates that an activated TGFβ pathway may be a shared mechanism of neurodegeneration in familial and sporadic ALS MNs. In support of this hypothesis, sporadic ALS MNs display elevated levels of phosphorylated SMAD2 in their nuclei compared with their healthy counterparts (Nakamura et al., 2008). Previous studies have postulated that elevated levels of TGFβ signaling arising from ALS astrocytes or muscle could drive MN death (Endo et al., 2015; Gonzalez et al., 2017; Si et al., 2015). Our study indicates that MNs themselves could be the source of TGFβ. Whether astrocytes secrete TGFβ independently or in response to MNs remains to be seen.

How does an activated TGFβ contribute to MN dysfunction and death? Analysis of TFs associated with TGFβ signaling provide insights into the underlying mechanism. The negative regulons of *SMAD2*, *TGIF1*, and *TGIF2* were enriched for genes involved in transmission across chemical synapses (adjusted p value <0.01), while the positive regulons of the TFs *TGIF2* and *ZFP36L1* were enriched for genes involved in mitotic cell cycle (adjusted p value <0.01). Reactivation of the cell cycle in post-mitotic neurons leads to activation of apoptotic pathways (Kruman et al., 2004). We observed upregulation of cell cycle genes in our SOD1 ipMN single-cell data, in accordance with our previous study (Bhinge et al., 2017). It must be noted that activation of cell cycle in our mitomycin-treated cultures could also lead to apoptosis. Our experimental system cannot distinguish between the two possibilities. However, if this was the case, we would expect to see a proportion of cells display strong KI67 nuclear signal. Absence of nuclear KI67 staining cells makes this possibility unlikely. Additionally, our previous work showed that inhibition of the cell cycle pathway does not lead to enhanced survival in our SOD1 ipMNs (Bhinge et al., 2017). This suggests that, as opposed to a full re-entry into mitosis, degenerating neurons display upregulation of cell cycle genes. The observed upregulation of genes related to the cell cycle pathway could be a downstream effect of other processes that drive the observed neurodegeneration. However, further experimentation would be required to distinguish between the two possibilities.

Our network analysis indicates that developmental gene expression programs may be reactivated and neuronal programs inhibited in SOD1 ipMNs. A recent study found reactivation of de-differentiation pathways and inhibition of mature neuronal programs in neurons transdifferentiated from Alzheimer disease patient fibroblasts (Mertens et al.,

2021). Thus reactivation of developmental programs may contribute to neuronal dysfunction in age-onset neurodegenerative disorders. This could contribute to downregulation of synaptic genes in diseased MNs, eventually leading to the synaptic collapse as well as drive upregulation of cell-cycle-related genes in degenerating neurons. We also observed activation of WNT signaling in the SOD1 ipMNs (pink module in this study). Our previous study had shown that an activated WNT pathway can drive neurodegeneration in SOD1 ipMNs (Bhinge et al., 2017). WNT activation is an early and essential event in the patterning of neural progenitors toward an MN fate (Maury et al., 2015). However, whether reactivation of developmental programs is the cause or a downstream effect of TGFβ or WNT activation needs further investigation. A limitation of our study is the analysis of the SOD1 neurons at a single time point, which cannot distinguish between reactivation of developmental programs or their failure to be completely suppressed.

Differential neuronal susceptibility has been recognized in ALS, with oculomotor neurons being relatively resistant to neurodegeneration (Hedlund et al., 2010). However, whether spinal interneurons are equally susceptible to neurodegeneration in ALS is unclear. Our results indicate that our iPSC-derived SOD1 V1 ipINs share many of the dysregulated pathways observed in the SOD1 ipMNs. However, genes involved in oxidative phosphorylation or the respiratory electron transport seem to be unaffected in the SOD1 V1 ipINs, while these were significantly downregulated in SOD1 ipMNs. Given the high metabolic demands of MNs, perturbation of mitochondrial pathways could make these neurons more susceptible to degeneration than V1 INs. On the other hand, SOD1 V1 ipINs also display upregulation of the same gene expression programs as observed in MNs, although to a weaker extent. This would suggest that SOD1 V1 ipINs might display survival deficits but may be more resistant to degeneration than MNs.

Conclusions

The underlying cause of MN degeneration in ALS is very likely to be multi-factorial with multiple drivers collaborating to cause MN demise. We have identified that dysregulation of TFs that disrupt MN homeostasis are major contributors to death in SOD1 ALS ipMNs. Our results display the power of applying network analysis with single-cell transcriptomics to iPSC-based neurodegenerative models to uncover drivers of MN degeneration in ALS.

EXPERIMENTAL PROCEDURES

Human iPSC culture

ALS patient-derived iPSCs bearing SOD1 E100G/+ (ND35662) mutation and the genome edited isogenic control iPSCs were maintained as colonies on human embryonic stem cell-qualified Matrigel (Corning) in mTeSR (StemCell Technologies). Colonies were routinely passaged in a 1:6 split using Dispase. Mycoplasma testing was conducted regularly to rule out mycoplasma contamination of cultures.

Differential gene expression analysis

We used DESeq2 with parameters optimized for single-cell data analysis using the command: DESeq (dds, test = "Wald", sfType = "poscounts", minReplicatesForReplace = Inf, useT = T, minmu = 1×10^{-6}). The design used for the analysis was batch + sample, where batch indicated the replicate and sample indicated the genotype.

WGCNA

k-NN smoothened normalized read counts were used to build a coexpression network. The coexpression network was constructed with WGCNA using a soft thresholding power of 6 using the signed-hybrid approach. Modules in the network were identified using the cutTreeDynamic function with a minimum module size of 50. Modules were merged if their eigengene correlation coefficients were ≥ 0.75. Pearson's correlation was used to assess associations between module eigengenes and disease state or neuronal subtypes. p values were corrected using the method of Benjamini and Hochberg, and correlations with an adjusted p value <0.01 were deemed significant. GO enrichment analysis of disease associated modules was carried out using the anRichment R package.

MRA

The RTN package was used to implement the ARACNE algorithm. Details are provided in Supplemental experimental procedures.

Data and code availability

The raw data have been submitted to ArrayExpress with accession number E-MTAB-7353.

SUPPLEMENTAL INFORMATION

Supplemental information can be found online at https://doi.org/10.1016/j.stemcr.2021.10.010.

AUTHORS CONTRIBUTIONS

A.B. conceptualized the study. A.B. and L.W.S. provided funding for the project. A.B., S.C.N., S.H., and L.O.G. designed and conducted the experiments. A.B., P.T., R.A., and C.R.G.W. analyzed the data. All authors contributed toward interpreting the data and writing the manuscript.

DECLARATION OF INTERESTS

The authors declare no competing interests.

ACKNOWLEDGMENTS

We thank the sequencing center at the Genome Institute of Singapore for help with Illumina sequencing. This work was generously supported by the Wellcome Trust Institutional Strategic Support Award (WT204909MA) and the Joint Council Office ASTAR, Singapore. A.B. is funded by an AMS Springboard Award

(SBF003\1182), an MRC grant (MR/T033029/1), and University of Exeter, U.K. start-up funds. R.A. is supported by a UKRI EPSRC Innovation Fellowship. C.R.G.W. is supported by the BBSRC-funded South West Biosciences Doctoral Training Partnership (BB/J014400/1; BB/M009122/1).

Received: March 9, 2021
Revised: October 17, 2021
Accepted: October 18, 2021
Published: November 11, 2021

REFERENCES

Basso, K., Margolin, A.A., Stolovitzky, G., Klein, U., Dalla-Favera, R., and Califano, A. (2005). Reverse engineering of regulatory networks in human B cells. Nat. Genet. *37*, 382–390.

Becht, E., McInnes, L., Healy, J., Dutertre, C.A., Kwok, I.W.H., Ng, L.G., Ginhoux, F., and Newell, E.W. (2018). Dimensionality reduction for visualizing single-cell data using UMAP. Nat. Biotechnol. https://doi.org/10.1038/nbt.4314.

Bhinge, A., Namboori, S.C., Zhang, X., VanDongen, A.M.J., and Stanton, L.W. (2017). Genetic correction of SOD1 mutant iPSCs reveals ERK and JNK activated AP1 as a driver of neurodegeneration in amyotrophic lateral sclerosis. Stem Cell Rep. *8*, 856–869.

Bilsland, L.G., Sahai, E., Kelly, G., Golding, M., Greensmith, L., and Schiavo, G. (2010). Deficits in axonal transport precede ALS symptoms in vivo. Proc. Natl. Acad. Sci. U. S. A. *107*, 20523–20528.

Brown, R.H., Jr., and Al-Chalabi, A. (2017). Amyotrophic lateral sclerosis. N. Engl. J. Med. *377*, 1602.

Cao, J., Spielmann, M., Qiu, X., Huang, X., Ibrahim, D.M., Hill, A.J., Zhang, F., Mundlos, S., Christiansen, L., Steemers, F.J., et al. (2019). The single-cell transcriptional landscape of mammalian organogenesis. Nature *566*, 496–502.

Dafinca, R., Barbagallo, P., Farrimond, L., Candalija, A., Scaber, J., Ababneh, N.A., Sathyaprakash, C., Vowles, J., Cowley, S.A., and Talbot, K. (2020). Impairment of mitochondrial calcium buffering links mutations in C9ORF72 and TARDBP in iPS-derived motor neurons from patients with ALS/FTD. Stem Cell Rep. *14*, 892–908.

De Santis, R., Santini, L., Colantoni, A., Peruzzi, G., de Turris, V., Alfano, V., Bozzoni, I., and Rosa, A. (2017). FUS mutant human motoneurons display altered transcriptome and microRNA pathways with implications for ALS pathogenesis. Stem Cell Rep. *9*, 1450–1462.

Endo, F., Komine, O., Fujimori-Tonou, N., Katsuno, M., Jin, S., Watanabe, S., Sobue, G., Dezawa, M., Wyss-Coray, T., and Yamanaka, K. (2015). Astrocyte-derived TGF-beta1 accelerates disease progression in ALS mice by interfering with the neuroprotective functions of microglia and T cells. Cell Rep. *11*, 592–604.

Fischer, L.R., Culver, D.G., Tennant, P., Davis, A.A., Wang, M., Castellano-Sanchez, A., Khan, J., Polak, M.A., and Glass, J.D. (2004). Amyotrophic lateral sclerosis is a distal axonopathy: evidence in mice and man. Exp. Neurol. *185*, 232–240.

Fletcher, M.N., Castro, M.A., Wang, X., de Santiago, I., O'Reilly, M., Chin, S.F., Rueda, O.M., Caldas, C., Ponder, B.A., Markowetz, F., et al. (2013). Master regulators of FGFR2 signalling and breast cancer risk. Nat. Commun. *4*, 2464.

Fujimori, K., Ishikawa, M., Otomo, A., Atsuta, N., Nakamura, R., Akiyama, T., Hadano, S., Aoki, M., Saya, H., Sobue, G., et al. (2018). Modeling sporadic ALS in iPSC-derived motor neurons identifies a potential therapeutic agent. Nat. Med. *24*, 1579–1589.

Garone, M.G., Birsa, N., Rosito, M., Salaris, F., Mochi, M., de Turris, V., Nair, R.R., Cunningham, T.J., Fisher, E.M.C., Morlando, M., et al. (2021). ALS-related FUS mutations alter axon growth in motoneurons and affect HuD/ELAVL4 and FMRP activity. Commun. Biol. *4*, 1025.

Gonzalez, D., Contreras, O., Rebolledo, D.L., Espinoza, J.P., van Zundert, B., and Brandan, E. (2017). ALS skeletal muscle shows enhanced TGF-beta signaling, fibrosis and induction of fibro/adipogenic progenitor markers. PLoS One *12*, e0177649.

Hall, C.E., Yao, Z., Choi, M., Tyzack, G.E., Serio, A., Luisier, R., Harley, J., Preza, E., Arber, C., Crisp, S.J., et al. (2017). Progressive motor neuron pathology and the role of astrocytes in a human stem cell model of VCP-related ALS. Cell Rep. *19*, 1739–1749.

Hardiman, O., Al-Chalabi, A., Chio, A., Corr, E.M., Logroscino, G., Robberecht, W., Shaw, P.J., Simmons, Z., and van den Berg, L.H. (2017). Amyotrophic lateral sclerosis. Nat. Rev. Dis. Primers *3*, 17085.

Hedlund, E., Karlsson, M., Osborn, T., Ludwig, W., and Isacson, O. (2010). Global gene expression profiling of somatic motor neuron populations with different vulnerability identify molecules and pathways of degeneration and protection. Brain *133*, 2313–2330.

Ho, R., Sances, S., Gowing, G., Amoroso, M.W., O'Rourke, J.G., Sahabian, A., Wichterle, H., Baloh, R.H., Sareen, D., and Svendsen, C.N. (2016). ALS disrupts spinal motor neuron maturation and aging pathways within gene co-expression networks. Nat. Neurosci. *19*, 1256–1267.

Ho, R., Workman, M.J., Mathkar, P., Wu, K., Kim, K.J., O'Rourke, J.G., Kellogg, M., Monte, V., Banuelos, M.G., Aladesuyi, O., et al. (2020). Single-cell RNA-seq analysis of human iPSC-derived motor neurons resolves early and predictive ALS signatures. BioRxiv https://doi.org/10.1101/2020.04.27.064584.

Ikiz, B., Alvarez, M.J., Re, D.B., Le Verche, V., Politi, K., Lotti, F., Phani, S., Pradhan, R., Yu, C., Croft, G.F., et al. (2015). The regulatory machinery of neurodegeneration in in vitro models of amyotrophic lateral sclerosis. Cell Rep. *12*, 335–345.

Kanehisa, M., and Goto, S. (2000). KEGG: Kyoto Encyclopedia of Genes and Genomes. Nucleic Acids Res. *28*, 27–30.

Kiskinis, E., Sandoe, J., Williams, L.A., Boulting, G.L., Moccia, R., Wainger, B.J., Han, S., Peng, T., Thams, S., Mikkilineni, S., et al. (2014). Pathways disrupted in human ALS motor neurons identified through genetic correction of mutant SOD1. Cell Stem Cell *14*, 781–795.

Kong, J., and Xu, Z. (1998). Massive mitochondrial degeneration in motor neurons triggers the onset of amyotrophic lateral sclerosis in mice expressing a mutant SOD1. J. Neurosci. *18*, 3241–3250.

Krach, F., Batra, R., Wheeler, E.C., Vu, A.Q., Wang, R., Hutt, K., Rabin, S.J., Baughn, M.W., Libby, R.T., Diaz-Garcia, S., et al. (2018). Transcriptome-pathology correlation identifies interplay between TDP-43 and the expression of its kinase CK1E in sporadic ALS. Acta Neuropathol. *136*, 405–423.

Kruman, , II, Wersto, R.P., Cardozo-Pelaez, F., Smilenov, L., Chan, S.L., Chrest, F.J., Emokpae, R., Jr., Gorospe, M., and Mattson, M.P.

(2004). Cell cycle activation linked to neuronal cell death initiated by DNA damage. Neuron *41*, 549–561.

Landrum, M.J., Chitipiralla, S., Brown, G.R., Chen, C., Gu, B., Hart, J., Hoffman, D., Jang, W., Kaur, K., Liu, C., et al. (2020). ClinVar: improvements to accessing data. Nucleic Acids Res. *48*, D835–D844.

Langfelder, P., and Horvath, S. (2008). WGCNA: an R package for weighted correlation network analysis. BMC Bioinform. *9*, 559.

Lenzi, J., De Santis, R., de Turris, V., Morlando, M., Laneve, P., Calvo, A., Caliendo, V., Chio, A., Rosa, A., and Bozzoni, I. (2015). ALS mutant FUS proteins are recruited into stress granules in induced pluripotent stem cell-derived motoneurons. Dis. Model. Mech. *8*, 755–766.

Liberzon, A., Birger, C., Thorvaldsdottir, H., Ghandi, M., Mesirov, J.P., and Tamayo, P. (2015). The molecular signatures database (MSigDB) hallmark gene set collection. Cell Syst. *1*, 417–425.

Liu, W., Venugopal, S., Majid, S., Ahn, I.S., Diamante, G., Hong, J., Yang, X., and Chandler, S.H. (2020). Single-cell RNA-seq analysis of the brainstem of mutant SOD1 mice reveals perturbed cell types and pathways of amyotrophic lateral sclerosis. Neurobiol. Dis. *141*, 104877.

Lopez-Gonzalez, R., Lu, Y., Gendron, T.F., Karydas, A., Tran, H., Yang, D., Petrucelli, L., Miller, B.L., Almeida, S., and Gao, F.B. (2016). Poly(GR) in C9ORF72-related ALS/FTD compromises mitochondrial function and increases oxidative stress and DNA damage in iPSC-derived motor neurons. Neuron *92*, 383–391.

Luisier, R., Tyzack, G.E., Hall, C.E., Mitchell, J.S., Devine, H., Taha, D.M., Malik, B., Meyer, I., Greensmith, L., Newcombe, J., et al. (2018). Intron retention and nuclear loss of SFPQ are molecular hallmarks of ALS. Nat. Commun. *9*, 2010.

Martin-Mateos, R., De Assuncao, T.M., Arab, J.P., Jalan-Sakrikar, N., Yaqoob, U., Greuter, T., Verma, V.K., Mathison, A.J., Cao, S., Lomberk, G., et al. (2019). Enhancer of zeste homologue 2 inhibition attenuates TGF-beta dependent hepatic stellate cell activation and liver fibrosis. Cell Mol. Gastroenterol. Hepatol. *7*, 197–209.

Massague, J. (2012). TGFbeta signalling in context. Nat. Rev. Mol. Cell Biol. *13*, 616–630.

Maury, Y., Come, J., Piskorowski, R.A., Salah-Mohellibi, N., Chevaleyre, V., Peschanski, M., Martinat, C., and Nedelec, S. (2015). Combinatorial analysis of developmental cues efficiently converts human pluripotent stem cells into multiple neuronal subtypes. Nat. Biotechnol. *33*, 89–96.

Mehta, A.R., Gregory, J.M., Dando, O., Carter, R.N., Burr, K., Nanda, J., Story, D., McDade, K., Smith, C., Morton, N.M., et al. (2021). Mitochondrial bioenergetic deficits in C9orf72 amyotrophic lateral sclerosis motor neurons cause dysfunctional axonal homeostasis. Acta Neuropathol. *141*, 257–279.

Mertens, J., Herdy, J.R., Traxler, L., Schafer, S.T., Schlachetzki, J.C.M., Bohnke, L., Reid, D.A., Lee, H., Zangwill, D., Fernandes, D.P., et al. (2021). Age-dependent instability of mature neuronal fate in induced neurons from Alzheimer's patients. Cell Stem Cell *28*, 1533–1548.

Nakamura, M., Ito, H., Wate, R., Nakano, S., Hirano, A., and Kusaka, H. (2008). Phosphorylated Smad2/3 immunoreactivity in sporadic and familial amyotrophic lateral sclerosis and its mouse model. Acta Neuropathol. *115*, 327–334.

Nakashima, H., Tsujimura, K., Irie, K., Ishizu, M., Pan, M., Kameda, T., and Nakashima, K. (2018). Canonical TGF-beta signaling negatively regulates neuronal morphogenesis through TGIF/Smad complex-mediated CRMP2 suppression. J. Neurosci. *38*, 4791–4810.

Nardo, G., Iennaco, R., Fusi, N., Heath, P.R., Marino, M., Trolese, M.C., Ferraiuolo, L., Lawrence, N., Shaw, P.J., and Bendotti, C. (2013). Transcriptomic indices of fast and slow disease progression in two mouse models of amyotrophic lateral sclerosis. Brain *136*, 3305–3332.

Nichterwitz, S., Chen, G., Aguila Benitez, J., Yilmaz, M., Storvall, H., Cao, M., Sandberg, R., Deng, Q., and Hedlund, E. (2016). Laser capture microscopy coupled with Smart-seq2 for precise spatial transcriptomic profiling. Nat. Commun. *7*, 12139.

Pastar, I., Stojadinovic, O., Krzyzanowska, A., Barrientos, S., Stuelten, C., Zimmerman, K., Blumenberg, M., Brem, H., and Tomic-Canic, M. (2010). Attenuation of the transforming growth factor beta-signaling pathway in chronic venous ulcers. Mol. Med. *16*, 92–101.

Philippidou, P., and Dasen, J.S. (2013). Hox genes: choreographers in neural development, architects of circuit organization. Neuron *80*, 12–34.

Rabin, S.J., Kim, J.M., Baughn, M., Libby, R.T., Kim, Y.J., Fan, Y., Libby, R.T., La Spada, A., Stone, B., and Ravits, J. (2010). Sporadic ALS has compartment-specific aberrant exon splicing and altered cell-matrix adhesion biology. Hum Mol Genet *19*, 313–328.

Rayon, T., Stamataki, D., Perez-Carrasco, R., Garcia-Perez, L., Barrington, C., Melchionda, M., Exelby, K., Tybulewicz, V., Fisher, W.M.C., and Briscoe, J. (2019). Species-specific developmental timing is associated with global differences in protein stability in mouse and human. BioRxiv https://doi.org/10.1101/2019.12.29.889543.

Selvaraj, B.T., Livesey, M.R., Zhao, C., Gregory, J.M., James, O.T., Cleary, E.M., Chouhan, A.K., Gane, A.B., Perkins, E.M., Dando, O., et al. (2018). C9ORF72 repeat expansion causes vulnerability of motor neurons to Ca(2+)-permeable AMPA receptor-mediated excitotoxicity. Nat. Commun. *9*, 347.

Si, Y., Kim, S., Cui, X., Zheng, L., Oh, S.J., Anderson, T., AlSharabati, M., Kazamel, M., Volpicelli-Daley, L., Bamman, M.M., et al. (2015). Transforming growth factor beta (TGF-beta) is a muscle biomarker of disease progression in ALS and correlates with Smad expression. PLoS One *10*, e0138425.

Steg, L.C., Shireby, G.L., Imm, J., Davies, J.P., Franklin, A., Flynn, R., Namboori, S.C., Bhinge, A., Jeffries, A.R., Burrage, J., et al. (2021). Novel epigenetic clock for fetal brain development predicts prenatal age for cellular stem cell models and derived neurons. Mol. Brain *14*, 98.

Sweeney, L.B., Bikoff, J.B., Gabitto, M.I., Brenner-Morton, S., Baek, M., Yang, J.H., Tabak, E.G., Dasen, J.S., Kintner, C.R., and Jessell, T.M. (2018). Origin and segmental diversity of spinal inhibitory interneurons. Neuron *97*, 341–355.e343.

Tesseur, I., Zou, K., Esposito, L., Bard, F., Berber, E., Can, J.V., Lin, A.H., Crews, L., Tremblay, P., Mathews, P., et al. (2006). Deficiency in neuronal TGF-beta signaling promotes neurodegeneration and Alzheimer's pathology. J. Clin. Invest. *116*, 3060–3069.

Wagner, F., Yan, Y., and Yanai, I. (2018). K-nearest neighbor smoothing for high-throughput single-cell RNA-seq data. bioRxiv https://doi.org/10.1101/217737.

Wang, J., Jenjaroenpun, P., Bhinge, A., Angarica, V.E., Del Sol, A., Nookaew, I., Kuznetsov, V.A., and Stanton, L.W. (2017). Single-cell gene expression analysis reveals regulators of distinct cell subpopulations among developing human neurons. Genome Res. *27*, 1783–1794.

Weina, K., Wu, H., Knappe, N., Orouji, E., Novak, D., Bernhardt, M., Huser, L., Larribere, L., Umansky, V., Gebhardt, C., et al. (2016). TGF-beta induces SOX2 expression in a time-dependent manner in human melanoma cells. Pigment Cell Melanoma Res. *29*, 453–458.

Stem Cell Reports, Volume *16*

Supplemental Information

Single-cell transcriptomics identifies master regulators of neurodegeneration in SOD1 ALS iPSC-derived motor neurons

Seema C. Namboori, Patricia Thomas, Ryan Ames, Sophie Hawkins, Lawrence O. Garrett, Craig R.G. Willis, Alessandro Rosa, Lawrence W. Stanton, and Akshay Bhinge

Supplementary methods

Differentiation of iPSC into spinal motor neurons

iPSC were plated as colonies onto matrigel and differentiated by treatment with neuronal differentiation media (DMEM/F12:Neurobasal in a 1:1 ratio, HEPES 10mM, N2 supplement 1%, B27 supplement 1%, L-glutamine 1%, ascorbic acid 5uM, insulin 20ug/ml) supplemented with SB431542 (40uM), CHIR9921 (3uM) and LDN8312 (0.2uM) from day 0 till day 4. Cells were caudalized by treatment with 0.1uM retinoic acid starting at day 2 and ventralized with 1uM purmorphamine starting at day 4 and continued till day 10. At day 10, progenitors were re-plated onto poly-D-lysine/laminin coated wells and differentiated by treating the cells with N2B27 media supplemented with BDNF 20ug/ml, GDNF 10ug/ml and DAPT 10uM. DAPT treatment was stopped at day 14 and neuronal cultures were pulsed with mitomycin at a dose of 10ug/ml for 1 hour to prevent further proliferation of any undifferentiated progenitors. Neuronal cultures were maintained by changing media every other day.

Immunofluorescence

Cells were fixed with 4% paraformaldehyde, permeabilized with ice-cold methanol for 5 minutes and washed with PBS containing 10% serum for 1 hour at room temperature. Cells were incubated with primary antibodies (Table S1) diluted into PBS containing 10% serum and incubated overnight at $4^{o}C$. Next day, cells were washed and incubated with Alexa-fluor conjugated secondary antibodies (Molecular probes) for 45 minutes at room temperature and nuclei were stained with Hoechst 33542 (Molecular probes). Images were obtained in an automated fashion on the ImageXpress Pico (Molecular devices). ISL1+ nuclei identification and p-SMAD2 nuclear signal was quantified in an automated fashion using the imageXpress software. Threshold intensities were maintained the same across wells.

Quantitative RT-PCR

Total RNA was extracted with the miRNeasy kit (Qiagen) and reverse transcribed using random hexamers and the HighCapacity reverse transcription system from Applied

Biosystems. Quantitative PCR was performed using the SYBR GREEN PCR Master Mix from Applied Biosystems. The target gene mRNA expression was normalized to the expression of two housekeeping genes (HPRT1 and RPL13), and relative mRNA fold changes were calculated by the ΔΔCt method. Primer sequences are included in Table S2.

MN survival assay

MNs were differentiated from SOD1 E100G and the isogenic control iPSC as described above in 96-well optically clear tissue culture plates. Day 30 cultures were fixed and stained for ISL1 to assess MN counts. A separate plate cultured under the same condition was allowed to proceed till day 44, when it was fixed and stained for ISL1. Nuclei were stained using Hoechst 33342. MN counts at day 40 were compared with day 30 to assess MN survival. We performed three independent differentiations with two technical wells used per replicate. Data from the technical wells was pooled to generate counts for each replicate.

Cell survival assays in response to TGFβ perturbation

We found that the process of immunostaining occasionally resulted in neuronal detachment leading to underestimates of neuronal counts. Hence, cultures that showed neuronal detachment were discarded. We realized that a better approach was to treat live neurons with the cell-permeable nuclear dye Hoechst 33342 and image the same well at different time points. Given that almost 80% of the cells in culture were motor neurons, nuclei counts were expected to closely approximate MN counts. Neuronal cultures at day 30 were treated with Hoechst 33542 (0.25 ug/ml) for 45 minutes and media was replaced with standard neuronal culture media as described above. Nuclear stained cultures were analysed by live imaging in the DAPI channel at day 30 and again at day 40. This allowed us to assess nuclear counts for the same well at the two different time points. Day 40 counts were compared with day 30 counts to assess percentage loss of neurons using 3 independent differentiations with two wells as technical replicates per treatment per differentiation. SB431542 was dissolved in DMSO. Hence, a DMSO only treatment was used as control for the TGFβ inhibition experiments. The final concentration of DMSO was maintained at 0.1%. TGFβ1 was

resuspended in PBS and we used neurons treated with PBS as control. Apoptosis was estimated in independent neuronal cultures using the Promega RealTime Glo apoptosis assay according to the manufacturer's instructions.

Single-cell capture and library preparation

Single cells were captured using standard protocol of C1 single-cell auto prep system (Fluidigm). Two independent differentiations were set up a few days apart. At day 44, differentiated neuronal cultures were dissociated into single cells by Accutase and loaded onto the C1 chip. We used one chip per genotype per differentiation. Chips for each replicate (one for the SOD1 E100G and the other for the isogenic control) were loaded in parallel into two separate machines. We used a total of four chips labelled A, B, C and D. Chips A and B captured the isogenic control MNs while chips C and D captured the SOD1 E100G MNs. Post cell capture, each well of the chip was manually inspected to identify wells bearing single cells. Next, lysis, reverse transcription and PCR amplification of the cDNA was perform in an automated fashion within the C1 instrument. To prepare single-cell libraries, cDNA products from each single cell were harvested from C1 chip followed by concentration and quantification using PicoGreen dsDNA Assay kit. Sequencing libraries were generated using Illumina Nextera XT library preparation kit.

Read processing, mapping and quality control

Fastq files were processed using Salmon with a partial decoy index for human gene annotation GENCODE release 19 (Harrow et al. 2012). Transcript level counts were collapsed to generate read counts per gene using the tximport R package. This yielded 33,000 transcripts across 365 libraries. Only libraries deemed to be single cells were retained for further analysis (332 cells). A gene was deemed to be poorly expressed if it was present in less than 10 cells at a read count threshold of 2. The filtering process yielded 332 single cells and 14774 genes for further analysis. To remove poorly amplified RNA libraries, single cell libraries were subjected to a set of quality control criteria that included: 1) total mapped reads, 2) percentage mapped reads,

3) percentage of mitochondrial reads, 4) number of genes expressed. To identify cells that were outliers, we performed a PCA using these criteria (Fig. S1a). The PC1 vs PC2 map identified cells that were outliers with respect to the number and percentage of mapped reads while PC3 identified cells that displayed low number of expressed genes and high levels of mitochondrial reads (Fig. S1a). Cells deemed as outliers were removed from further analysis. After quality filtering, we retained a total of 323 high quality cells (163 cells for SOD1 and 160 cells for the control). Next, we removed lowly expressed genes (counts < 2) that were expressed in less than 10 cells. In summary, we obtained 323 cells that expressed 14774 genes in total with SOD1 cells expressing on average 7170 genes while the control dataset expressed 7745 genes with the overall distribution of the number of genes being similar between the two datasets (Fig. S1b). Additionally, other quality controls metrics including total number of reads, proportion of mapped reads, proportion of mitochondrial reads, duplicated sequences and GC content were similar across the SOD1 and isogenic control datasets (Fig. S1b). Differences between the isogenic control and ALS datasets for the proportion of mapped reads, mitochondrial reads and duplication rates were deemed significant by a t.test (Fig. S1b). However, the magnitude of the differences was small (0.68 vs 0.73 for the proportion of mapped reads, 21.7% vs 14.5% for the % mitochondrial mapped reads, 54.4% vs 58.8% for the duplication rate). We noted that the isogenic control cells from the replicate 2 displayed significantly higher percentage of mitochondrial mapped reads than replicate 1. Hence, it was deemed appropriate to control for this potential batch effect in the differential expression analysis. Each gene was classified based on whether it was protein coding, long non-coding, pseudogene or small nuclear/nucleolar RNA. We did not find any systematic difference in distribution of the gene classes expressed between the SOD1 and controls datasets (Fig. S1c). Further, principal component analysis using all expressed genes confirmed that our data did not show any batch effects arising from the independent differentiations or the capture plates (Fig. S1d). Finally, the PCA did not reveal any bias towards any specific dataset for the number of mapped reads, number of detected genes or mitochondrial genes (Fig. S1e).

Identifying genes affected by ambient RNA

Single cell dissociation and harvest protocols lead to lysis of cells that release their cellular mRNA into the mixture. This RNA termed "ambient mRNA" is released from dying cells and has been observed in the absence of cell capture in droplet based platforms(Angelidis et al. 2019). The ambient mRNAs typically arise from genes expressed at high levels. We observed that some of the microfluidic chambers that were marked as empty had generated RNA reads that mapped to human transcripts. It is possible that these chambers contained a cell that was missed by the human observer. But we decided to treat transcript counts arising from empty chambers as ambient mRNA. For each plate, we summed up the counts per gene across the empty chambers and estimated counts per million (cpm) reads for each gene. Next, we averaged the cpm values per gene across the two replicates plates for the healthy and ALS samples separately. We marked all genes at a cpm threshold above 100 as being affected by ambient RNA expression. We performed this filtering for the healthy and ALS samples individually to avoid excluding genes that were lowly expressed in either the healthy or the ALS samples. This resulted in a total of 1120 genes being marked as affected by the ambient mRNA. The ambient geneset was excluded from the clustering analysis to identify neural subtypes. Additionally, we excluded these genes from the sorted differentially expressed geneset used in the master regulator analysis (see below). This genelist has been included as Supplementary data file S1.

Identifying classifier genes

Neuron vs glia classifier gene set

We first identified genes differentially expressed between neurons and glia using a recently published gene expression dataset on purified human neurons, astrocytes and oligodendrocytes from frozen brain tissue(Zhang et al. 2016). Genes that displayed a fold change of at least 20 between neurons and astrocytes or oligodendrocytes were included for future analysis. Differential gene expression analysis identified 707 genes as differentially regulated between neurons versus glial cells. Gene ontology using DAVID(Huang da et al. 2009) on the differentially expressed gene set showed enrichment of specific functional

categories related to neuronal physiology. Categories related to neuron development such as GABAergic synapse and postsynaptic cell membrane were enriched in the neuron-activated genes while cell cycle and glial differentiation terms were deemed to be enriched in the downregulated genes confirming that our identified gene set was able to distinguish neurons and glia (Fig. S2a). Out of the 707 genes, 682 genes were expressed in our filtered single cell dataset. This list of genes was termed neuron_vs_nonneuron.

MN vs IN classifier gene set

To identify genesets that can differentiate between MNs and INs, we used the “knowledge matrix” defined previously to classify neurons isolated from embryonic mouse spinal cord into specific subtypes (Delile et al. 2019). This a binary matrix mined from published literature on key genes expressed in specific neuronal subtypes. We updated this matrix to include the following genes that are highly expressed in glial cells and progenitors: S100B, SOX9, PAX6, MKI67 and REST (Supplementary data file S2). In total, the matrix comprised of 52 genes and 14 cell types. Next, we extracted the expression data for the 52 genes from our single cell read counts for the healthy cells and binarized the counts for each gene using K-means clustering. We performed this analysis only on the healthy cells to avoid including any influence of the SOD1 mutation on marker gene expression. Our goal was to use this initial set of 52 genes to extract a wider gene set that can be used to classify the SOD1 and healthy data sets. The expression profile for each gene across the healthy cells was clustered using k=2 that separated the profile into low and high expression clusters. The high expression cluster was used to estimate a threshold for binarizing the gene expression vector (Methods). This resulted in a binary profile for the 52 markers gene for each cell. Each cell was now compared with each of the cell types in the knowledge matrix by estimating a jaccard coefficient (JC), which was used to classify a cell as a MN or IN. Due to the low number of cells, we did not distinguish between IN subtypes at this stage. To validate our classifier, we used it to identify cell types in an external dataset that include gene expression profiles of MNs, INs and progenitors purified using FACS from embryonic mouse spinal cord(Amin et al. 2015). We were able to classify all profiled cell types correctly into three main classes, namely MNs, INs, and non-neuronal cells

(Fig. S2b). We deployed our classifier to identify the top 25 cells (based on the JC) classified as MN or IN in our dataset. Gene expression of markers indicated that the classification was accurate (Fig. S2c). These cells were used to generate a differential expression list that could be used to distinguish MNs from INs based on their expression profile. This set of 600 genes, termed the MN_vs_IN geneset, was combined with our neuron_vs_nonneuron list described above. After filtering for genes affected by ambient RNA expression (Methods), we generated a list of 1060 unique classifier genes.

Differential expression analysis of MN sub-clusters

We noted that cluster 2 in Fig. 1f had lower number of SOD1 MNs compared to the isogenic controls. To ascertain that differences in the membership of SOD1 and control neurons was not driving the observed changes, we performed differential gene expression analysis of clusters 1 and 2 separately. We identified 129 genes as downregulated and 471 genes as upregulated in SOD1 ipMNs in cluster 1 at a p-value threshold of 0.01. Out of the 129 genes, 55 were also downregulated in the full dataset while the 305 genes overlapped between the upregulated genesets (Fig. S2e). Due to the low number of cells in the SOD1 set in cluster 2, we identified only 2 genes as significantly downregulated and 0 genes as upregulated in SOD1 ipMNs at an adjusted p-value threshold of 0.01. Applying a less stringent p-value threshold of 0.1 identified 137 genes as downregulated in SOD1 ipMNs, a number comparable with the 170 genes downregulated using all the ipMNs. Only 14 of the 137 genes overlapped with the downregulated genes identified using the full dataset though the overlap was statistically significant (Fig. S2f). At the p-value threshold of 0.1, only 8 genes were identified as upregulated in the cluster 2 set (Fig. S2f). Next, we performed pathway enrichment analysis of differentially expressed genesets in clusters 1 and 2 using GSEA (Fig. S2g, S2h). GSEA revealed downregulation of gene sets pertaining to mitochondrial gene expression in cluster 1 SOD1 MNs while mitochondrial respiratory transport and oxidative phosphorylation gene sets were downregulated in cluster 2 SOD1 MNs. Lowering the adjusted p-value threshold stringency of the GSEA to 0.1 revealed gene sets pertaining to axon guidance, neuron

projection and anterograde trans-synaptic transmission as downregulated in SOD1 MNs from the cluster 1 dataset but not in the cluster 2 dataset. Both datasets showed upregulation of the cell cycle pathway while terms related to mitosis, nonsense mediated decay and ribosome were upregulated in the cluster 1 MNs (Fig. S2g, S2h). These results indicate that differential membership in cluster 2 was not driving the observed differential expression in the full dataset.

kNN smoothing of single cell read counts

We adapted the algorithm described by Wagner et al(Wagner et al. 2018). Briefly, the read count smoothing algorithm worked as follows: 1) We started with a matrix of N cells X m genes where N=189 and m=14774 2) Count data was variance stabilized by taking the logarithm of the counts after adding 1 for each value in the single cell matrix. This was because, for our dataset, the log transformation worked better than the Freeman Tukey transform used in the original study. 3) Log transformed read counts were quantile normalized. 4) Euclidean distances between the cells were calculated using the first 8 principal components. The threshold of 8 was determined empirically using a scree plot. 4) For each cell, we calculated a weighted average of the read counts per gene between that cell and its k neighbours. The weights for the averaging are assigned as the inverse square root of the Euclidean distance between the cell and its neighbour. This ensured that neighbours that were far away in Euclidean space did not contribute as much to the final smoothened count than the nearest neigbours. The value of k is iteratively increased starting from 1 according to the following equation: $k = \min(2^{step} - 1, kmax)$; kmax = sqrt(N); step = 1:max_steps; max_steps = floor(log2(kmax + 1)); For example, in our study, for N=189, we get kmax=14 and max_steps=3.0. This results in 3 iterations where the value if k equals 1, 3 and 7 in each iteration successively. The final output is a matrix of counts that are quantile normalized and log transformed.

Estimating WGCNA module robustness

We estimated module robustness using a method detailed by Shannon et al(Shannon et al. 2016). The 189 neurons were sampled 50 times with replacement and modules were identified using the smoothened gene expression profiles for each sampling. The number of modules obtained per iteration ranged from 24-33 with a median of 29, which was close to the number obtained using all 189 neurons (26 modules). Each module in the original set of 26 modules identified using all 189 neurons was compared with all modules obtained in each iteration. Similarity between two modules A and B was defined as $(A \cap B)/\min(A, B)$ where $A \cap B$ indicates the number of genes common to A and B, while min(A,B) indicates the size of the smaller module between A and B(Shannon et al. 2016). We retained the maximum similarity score for each of the 26 modules per iteration. The final stability scores for each module were calculated as the average similarity scores across the 50 simulations.

Master regulator analysis

Smoothened counts for 189 neurons were used as input to the ARACNE algorithm to build a transcriptional network for 1137 TFs present in the dataset. TF annotations were obtained from AnimalTFDB. P-values for network edges were estimated from a pooled null distribution using 1000 permutations. Since we were asking ARACNE to evaluate interactions between 1137 TFs and 12550 target genes, a p-value threshold of 5e-8 would result in < 1 (1137*12550*5e-8) false positive edges to be included in the final network. At a p-value threshold of 5e-8, we identified a total of 1,255,493 edges between 1137 TFs and 12550 target genes with an average of 987 targets predicted per TF. The predicted targets of each TF were termed as the regulon. The regulon for each TF was classed as positive or negative based on the Pearson correlations. To identify master regulators, the differential gene expression between SOD1 and control ipMNs (after removing the ambient geneset) was used as a phenotype and sorted from most upregulated to most downregulated. The RTN package was used to conduct a GSEA like analysis to identify whether a TF regulon (positive or negative) was enriched towards one end of the sorted list of differentially expressed genes. P-values were

estimated based on 1000 permutations of the dataset and adjusted using the Benjamini Hochberg method. GO analysis of the regulons was carried out using the anRichment R package.

Analysis of publicly available ALS datasets

Normalized read count data for GSE54409 (human SOD1 A4V iPSC derived MNs purified using flow sorting based on the HB9 reporter)(Kiskinis et al. 2014) were downloaded from the gene expression omnibus(GEO). P-values were estimated by performing a t.test per gene. Read counts were averaged across replicates per gene and log2 transformed. Fold changes were estimated by subtracting the log2 counts for the ALS and isogenic controls: log2(ALS) – log2(control). Microarray expression values for GSE46298 (laser-capture microdissected MNs from spinal tissue obtained from the mouse SOD1 G93A ALS model)(Nardo et al. 2013) and GSE18920 (laser-capture microdissected MNs from spinal tissue obtained from sporadic ALS patients post-mortem)(Rabin et al. 2010) were downloaded from the gene expression omnibus. Expression values were background subtracted, normalized and log transformed using RMA from the affy R package(Gautier et al. 2004). Only genes with median expression values above background were included in the analysis. Probes mapping to the same gene were collapsed. Differential expression analysis was performed using the limma package in R(Ritchie et al. 2015). Differentially expressed genes for GSE76220 (laser-capture microdissected MNs from spinal tissue obtained from sporadic ALS patients post-mortem) (Krach et al. 2018) were obtained from the supplementary data for that study. This study had removed genes activated by the wound healing response by excluding genes with the highest principal component (PC1) eigengene values. We obtained both, the full (all genes) and the filtered (PC1 adjusted genes) datasets. Count data for GSE98920 (VCP R191Q, R155C) was obtained from GEO(Hall et al. 2017) while counts for the bulk C9ORF72 datasets were requested from the authors(Selvaraj et al. 2018). Both sequencing datasets were analyzed using DESeq2. For GSE98288, we only used counts from the control iPSC differentiations. P-

values for all datasets were corrected for multiple hypotheses using the Benjamini Hochberg procedure. Differentially expressed genes identified from our bulk RNA-seq analysis of SOD1 E100G iPSC derived MNs were obtained as described previously(Bhinge et al. 2017).

Count data for GSE140747(Rayon et al. 2019) (motor neuron development gene expression: D0-D15) and GSE98288(Hall et al. 2017; Luisier et al. 2018) (D21, D35 vs iPSC) was downloaded from the gene expression omnibus. We used DESeq2 to generate a list of genes differentially expressed between D0 neuromesodermal progenitors (NMPs) and D15 MNs. This list was sorted such that genes most upregulated in the NMPs were at the top while genes most downregulated in the NMPs were at the bottom. This process was repeated for time points D1 to D8 generating a set of differentially expressed ranked genelists for neural progenitors. By reversing the genelist D7 vs D15, we generated an additional ranked list of genes that had neuron-specific genes at the top of the list. Additionally, we also generated ranked gene expression profiles in immature (D21) and mature (D35) MNs differentiated from healthy iPSCs(Hall et al. 2017; Luisier et al. 2018). To sort genes, each gene was assigned a score = -1*log10(p-value) * sign(fold change). Genes were sorted based on this score in a decreasing manner so that the most significantly up regulated genes were assigned to the top of the list.

Supplementary figures

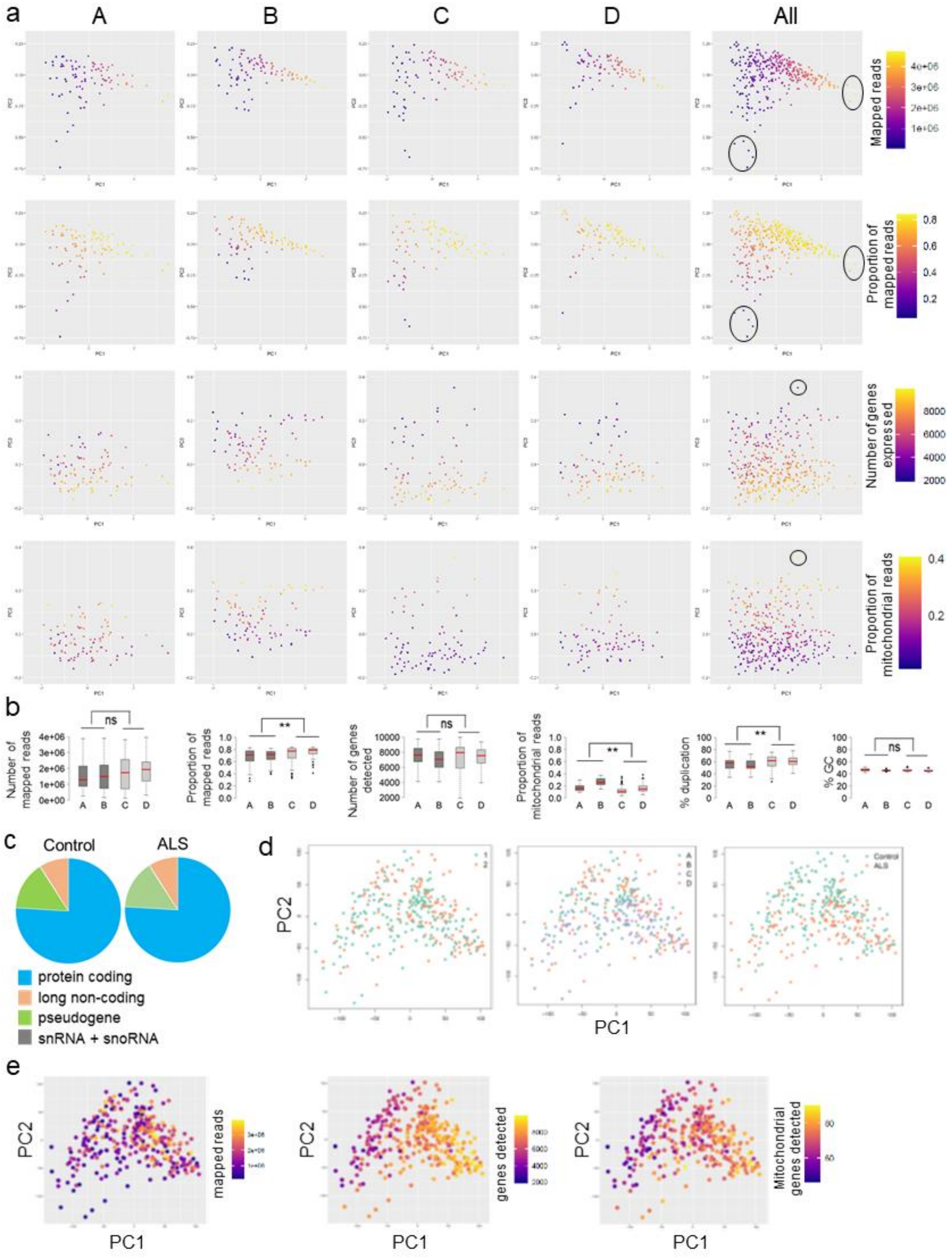

Fig. S1. Quality check of the single cell data

a) PCA plots displaying the different quality metrics used to filter cells. Each dot represents a cell. The black ellipses mark cells classified as outliers. Overall, nine cells were identified as outliers. b) Boxplots showing number of mapped reads, proportion of mapped reads, number of expressed genes, proportion of mitochondrial genes, percentage duplication and GC content in control and SOD1 datasets after removing outlier cells. P-values were estimated using a two-tailed Students's t.test by comparing the isogenic control dataset (cells from plates A, B) with the SOD1 dataset (cells from plates C, D). c) Pie chart showing the distribution of the expressed genes in different classes. d) PCA plots generated using all expressed genes show there is no batch effect between the datasets. Each dot represents a cell. Left panel: cells have been coloured based on the replicates. Middle panel: cells have been coloured based on which C1 fluidigm plate they were captured in. A: Control replicate 1, B: Control replicate 2, C: SOD1 replicate 1, D: SOD1 replicate 2. Right panel: Cells have been coloured based on genotype (i.e, isogenic control or ALS SOD1 E100G). e) PCA plots generated using all expressed genes showing distribution of mapped reads, proportion of mapped reads and number of mitochondrial genes detected.

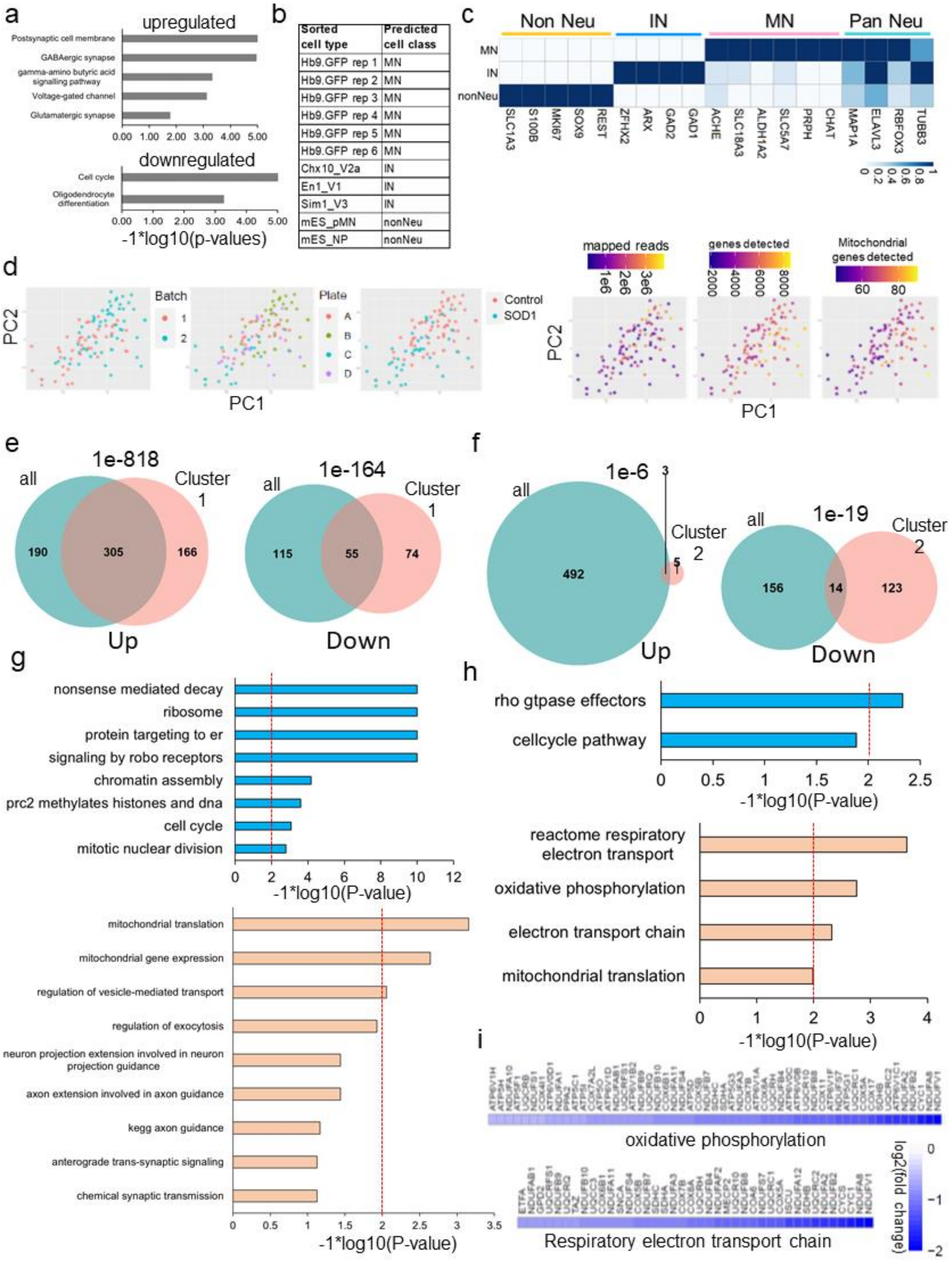

Fig. S2. Classification and differential gene expression of single cell data

a) GO enrichment analysis of neuronal vs glial classifier gene set. b) Validation of the classification process using an independent dataset (GSE75599). The classifier correctly identifies the three key classes: MNs, INs and non-neuronal cells. c) Classification of cells as MNs, INs, and non-neuronal cells based on the external dataset of marker genes. Normalized mean expression of neural markers across the three classes. Pan Neu: pan neuronal markers, Non Neu: non-neuronal markers. d) PCA plots generated using all expressed genes for the MN subset showing distribution of batch, plate, genotype, mapped reads, proportion of mapped reads and number of mitochondrial genes detected. e,f) Overlap between genes up or downregulated in SOD1 ipMNs in cluster 1 (e) or cluster 2 (f) and all ipMNs. Significance was estimated using the hypergeometric distribution. g,h) Pathways identified using gene set enrichment analysis (GSEA) on genes up (blue bars) or down (orange bars) regulated in SOD1 ipMNs in cluster 1 (g) and cluster 2 (h). p-values were adjusted using the Benjamini Hochberg procedure. i) Leading edge analysis reveals the core set of genes driving the observed enrichment scores in the GSEA for the terms “oxidative phosphorylation” and “respiratory electron transport chain”. The identified genes are nuclear-encoded mitochondrial transcripts. Color indicates log2 fold changes of SOD1 ipMNs vs control ipMNs.

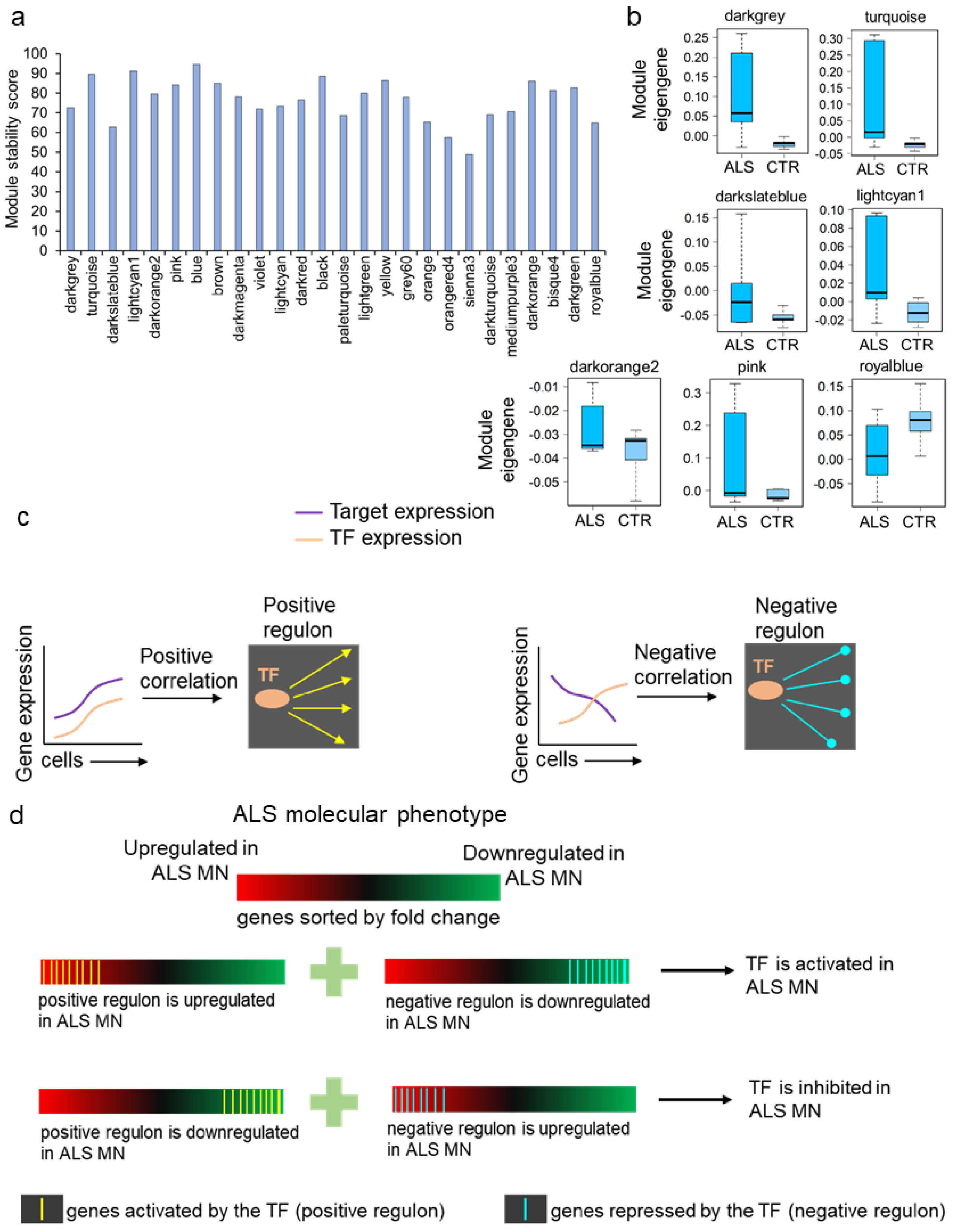

Fig. S3. Assessment of WGCNA identified modules

a) Bar plot showing stability scores for each module. Scores were estimated as described in Supplementary methods. b) Module eigengenes (the first principal component of the

expression of genes assigned to a module) were used to represent the overall expression of the module. Boxplots show the distribution of the module eigengene values between SOD1 and Control (CTR) ipMNs for the modules identified to be significantly associated with SOD1 ipMNs. Positively associated modules: darkgrey, turquoise, darkslateblue, lightcyan1, darkorange2, pink. Negatively associated module: royalblue. c) Generating a transcriptional network using ARACNE. The algorithm estimates pairwise mutual information (MI) scores between TFs and potential target genes, and estimates a significance threshold (p-value) based on a null distribution of scores. MI scores that pass this threshold are deemed valid TF-target pairs. The MI scores do not have a direction i.e. they are always positive. To estimate directionality of regulation, ARACNE estimates correlation between the TF and its target. A positive correlation indicates the TF activates the target while a negative correlation indicates that the TF represses the target. The positive targets of a TF are termed as the positive regulon while the negative targets are deemed the negative regulon. d) Master regulator analysis to identify drivers of SOD1 ipMN dysregulation. Genes differentially expressed in SOD1 ipMNs are sorted from most upregulated to most downregulated. The algorithm then estimates the enrichment of the positive and negative regulons of each TF across the sorted gene expression dataset. For a given TF, if the positive regulon is enriched in the upregulated end of the sorted genelist and the negative regulon is enriched in the downregulated end, the TF is assigned a positive score. If the inverse is true, the TF is assigned a negative score. TFs with significant positive scores are deemed to be activated in SOD1 ipMNs and are most likely to drive the SOD1 ipMN gene expression. On the other hand, TFs with significant negative scores are deemed to be inhibited in SOD1 ipMNs. The significance of a score is estimated using random simulations.

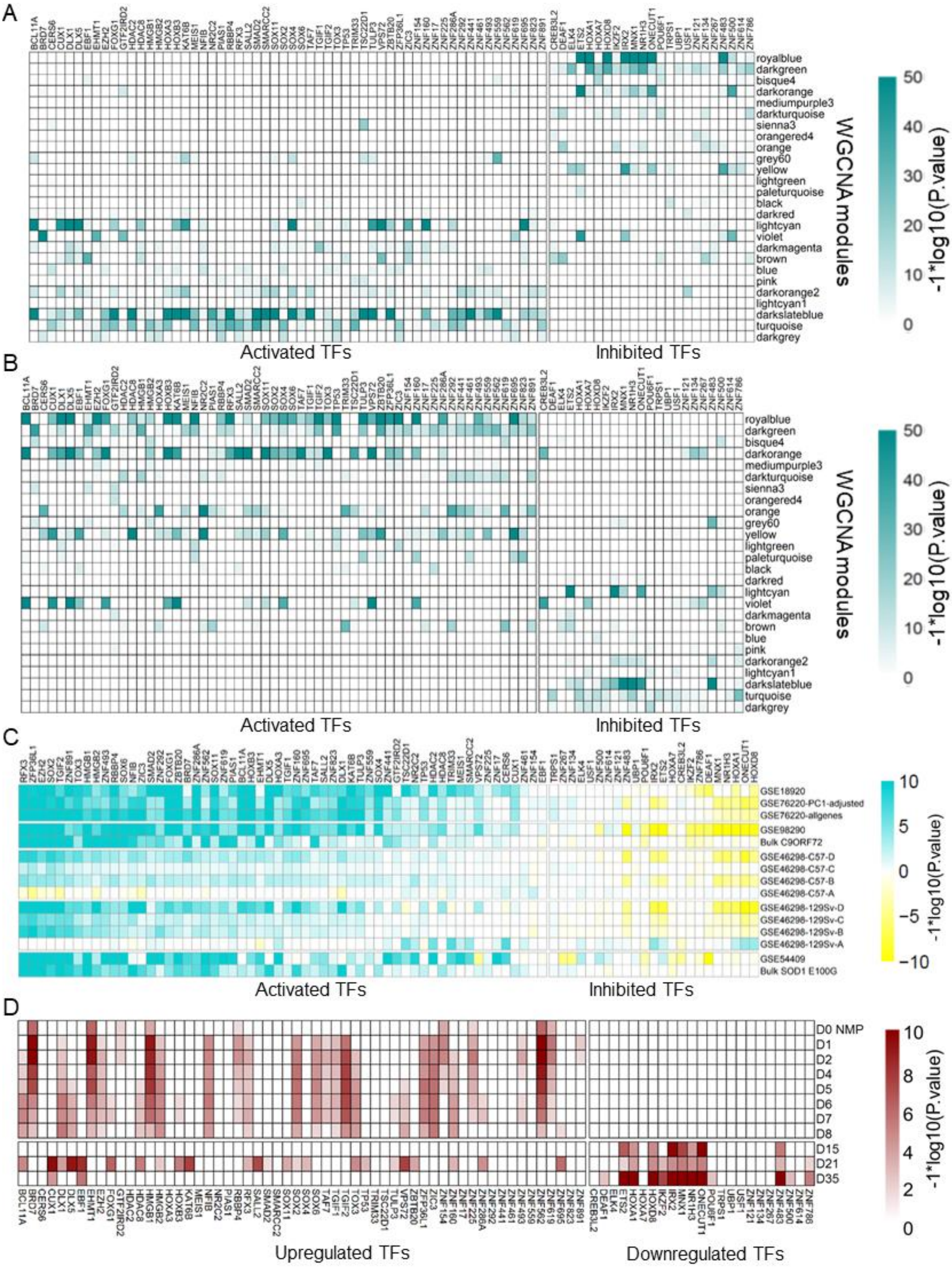

Fig. S4. Overlap analysis of TF regulons

a,b) Overlap between regulons of the 81 TFs identified as master regulators of SOD1 ipMN gene dysregulation and WGCNA modules. (a) analysis using the positive regulons of the TFs. (b) analysis using the negative regulons of the TFs. Significance of the overlap was estimated using a hypergeometric distribution. P-values were adjusted using the Benjamini Hochberg procedure, log transformed and plotted as a heatmap. c) Enrichment of the positive regulons of the 81 master regulator TFs in publicly available ALS datasets. The datasets are as described in Table 1. Positive regulons were used as input genesets for a GSEA performed on each dataset. Log transformed P-values were assigned the same sign as the GSEA enrichment scores and plotted as a heatmap. Green indicates that the regulon was activated while yellow indicates that the regulon was inhibited in the queried dataset of differentially expressed genes. d) Progenitor gene expression programs are reactivated in ALS MN. Enrichment of the positive regulons of the 81 master regulators identified by the MRA. Enrichment was estimated using a one-way GSEA where the positive regulons of each TF were used as genesets. The differentially expressed sorted progenitor and MN genelists were the same as those used in figure 3. Heatmap shows log transformed p-values.

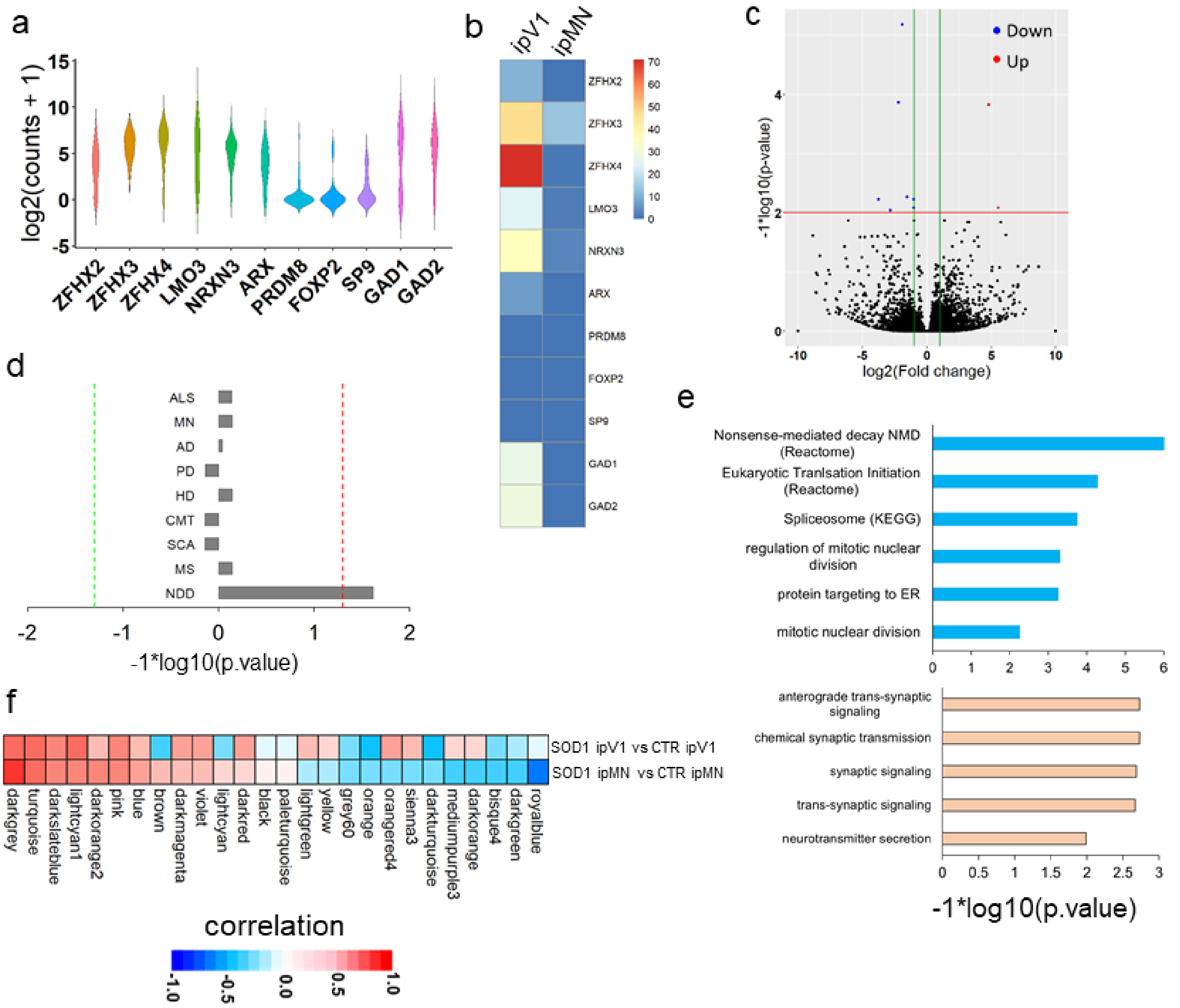

Fig. S5. Differential gene expression analysis of SOD1 V1 INs

a) Violin plot showing log2 transformed expression values of genes known to be expressed in V1 ipINs. b) Normalized mean expression of V1 markers between V1 ipINs and ipMNs. c) Volcano plot of genes differentially expressed in SOD1 V1 ipINs. Each dot represents a gene. Red: upregulated, blue: downregulated, black: unchanged genes. Horizontal red line indicates a p-value of 0.01. Vertical green lines indicate a log2(fold change) = 1. Fold changes >10 or <-10 were set 10 and -10 respectively. All p-values were adjusted using the Benjamini Hochberg procedure. d) Enrichment analysis of likely pathogenic variants associated with different diseases in genes upregulated in SOD1 V1 ipINs. Vertical axis shows terms used to search the ClinVar database to find associated pathogenic variants. ALS: Amyotrophic Lateral Sclerosis, MN: Motor neuron, AD: Alzheimers disease, PD: Parkinsons disease, HD: Huntingtons

disease, CMT: Charcot Marie Tooth, SCA: Spinocerebellar Ataxia, MS: Multiple Sclerosis, NDD: Neurodevelopment disorder. The red and green dashed lines indicate a p-value threshold of 0.05. Values on the right side of the axis indicate enrichment in upregulated genes while values on the left indicate enrichment in downregulated genes. e) Enrichment of GO terms in genes upregulated (blue bars) or downregulated (orange bars) in SOD1 V1 ipINs. The red dashed line indicates a p-value threshold of 0.01. f) Correlation analysis of WGCNA module eigengenes SOD1 V1 ipINs and ipMNs. Red: positive correlation, blue: negative correlation.

Table S1. Primary antibodies used for immunofluorescence. Related figures 1,5.

Target	Source	Catalog number	Dilution
ISL1	Abcam	ab8650	1:500
ISL1	Abcam	ab109517	1:500
MAP2	Abcam	ab11267	1:1000
NF-H	Sigma	N4142	1:1000
Phospho-SMAD2	Cell signalling technology	18338	1:200
KI-67	Cell signalling technology	9449	1:200

Table S2: Primer sequences used for qPCR analysis. Related to figure 5.

Gene	Forward primer	Reverse primer
TGIF1	GGATTGGCTGTATGAGCACCGT	GCCATCCTTTCTCAGCATGTCAG
TGIF2	CTGAGCCTTTCTGGACAGACCA	GGTCTTTGCCATCCTTCCGAAG
HMGA2	GAAGCCACTGGAGAAAAACGGC	GGCAGACTCTTGTGAGGATGTC
VIM	AGGCAAAGCAGGAGTCCACTGA	ATCTGGCGTTCCAGGGACTCAT
MYO10	CACTCTGCCGTATTTCCACAGC	TTTGTGGAGCCAGCCTTGCTTG
MEGF10	TGACTGCTTGCCTGGCTTCACA	GTTACAGGTTCCGTTGTTGGTGC
HES1	GGAAATGACAGTGAAGCACCTCC	GAAGCGGGTCACCTCGTTCATG
ITGA5	GCCGATTCACATCGCTCTCAAC	GTCTTCTCCACAGTCCAGCAAG
TGFBI	GGACATGCTCACTATCAACGGG	CTGTGGACACATCAGACTCTGC
TGFB1	GTGAGGTCCACGGAAACTGT	TGGCTGGTGCAAAGACATAG
ZFP36L1	CAGGATTCTCTCTCGGACCAG	CAGGCGTCTTGAGTTGTCCA
HPRT1	TGCTCGAGATGTGATGAAGG	AATCCAGCAGGTCAGCAAAG
RPL13	CCTGGAGGAGAAGAGGAAAGAGA	TTGAGGACCTCTGTGTATTTGTCAA

Supplementary datafile S1.xls

Genes affected by ambient expression.

Supplementary datafile S2.xls

Marker expression matrix to classify cell types.

Amin ND, Bai G, Klug JR, Bonanomi D, Pankratz MT, Gifford WD, Hinckley CA, Sternfeld MJ, Driscoll SP, Dominguez B et al. 2015. Loss of motoneuron-specific microRNA-218 causes systemic neuromuscular failure. *Science* **350**: 1525-1529.
Angelidis I, Simon LM, Fernandez IE, Strunz M, Mayr CH, Greiffo FR, Tsitsiridis G, Ansari M, Graf E, Strom TM et al. 2019. An atlas of the aging lung mapped by single cell transcriptomics and deep tissue proteomics. *Nat Commun* **10**: 963.
Bhinge A, Namboori SC, Zhang X, VanDongen AMJ, Stanton LW. 2017. Genetic Correction of SOD1 Mutant iPSCs Reveals ERK and JNK Activated AP1 as a Driver of Neurodegeneration in Amyotrophic Lateral Sclerosis. *Stem Cell Reports* **8**: 856-869.
Delile J, Rayon T, Melchionda M, Edwards A, Briscoe J, Sagner A. 2019. Single cell transcriptomics reveals spatial and temporal dynamics of gene expression in the developing mouse spinal cord. *Development* **146**.
Gautier L, Cope L, Bolstad BM, Irizarry RA. 2004. affy--analysis of Affymetrix GeneChip data at the probe level. *Bioinformatics* **20**: 307-315.
Hall CE, Yao Z, Choi M, Tyzack GE, Serio A, Luisier R, Harley J, Preza E, Arber C, Crisp SJ et al. 2017. Progressive Motor Neuron Pathology and the Role of Astrocytes in a Human Stem Cell Model of VCP-Related ALS. *Cell Rep* **19**: 1739-1749.
Harrow J, Frankish A, Gonzalez JM, Tapanari E, Diekhans M, Kokocinski F, Aken BL, Barrell D, Zadissa A, Searle S et al. 2012. GENCODE: the reference human genome annotation for The ENCODE Project. *Genome Res* **22**: 1760-1774.
Huang da W, Sherman BT, Lempicki RA. 2009. Bioinformatics enrichment tools: paths toward the comprehensive functional analysis of large gene lists. *Nucleic Acids Res* **37**: 1-13.
Kiskinis E, Sandoe J, Williams LA, Boulting GL, Moccia R, Wainger BJ, Han S, Peng T, Thams S, Mikkilineni S et al. 2014. Pathways disrupted in human ALS motor neurons identified through genetic correction of mutant SOD1. *Cell Stem Cell* **14**: 781-795.
Krach F, Batra R, Wheeler EC, Vu AQ, Wang R, Hutt K, Rabin SJ, Baughn MW, Libby RT, Diaz-Garcia S et al. 2018. Transcriptome-pathology correlation identifies interplay between TDP-43 and the expression of its kinase CK1E in sporadic ALS. *Acta Neuropathol* **136**: 405-423.
Luisier R, Tyzack GE, Hall CE, Mitchell JS, Devine H, Taha DM, Malik B, Meyer I, Greensmith L, Newcombe J et al. 2018. Intron retention and nuclear loss of SFPQ are molecular hallmarks of ALS. *Nat Commun* **9**: 2010.
Nardo G, Iennaco R, Fusi N, Heath PR, Marino M, Trolese MC, Ferraiuolo L, Lawrence N, Shaw PJ, Bendotti C. 2013. Transcriptomic indices of fast and slow disease progression in two mouse models of amyotrophic lateral sclerosis. *Brain* **136**: 3305-3332.
Rabin SJ, Kim JM, Baughn M, Libby RT, Kim YJ, Fan Y, Libby RT, La Spada A, Stone B, Ravits J. 2010. Sporadic ALS has compartment-specific aberrant exon splicing and altered cell-matrix adhesion biology. *Hum Mol Genet* **19**: 313-328.
Rayon T, Stamataki D, Perez-Carrasco R, Garcia-Perez L, Barrington C, Melchionda M, Exelby K, Tybulewicz V, Fisher WMC, Briscoe J. 2019. Species-specific developmental timing is associated with global differences in protein stability in mouse and human. *BioRxiv* doi:https://doi.org/10.1101/2019.12.29.889543.
Ritchie ME, Phipson B, Wu D, Hu Y, Law CW, Shi W, Smyth GK. 2015. limma powers differential expression analyses for RNA-sequencing and microarray studies. *Nucleic Acids Res* **43**: e47.
Selvaraj BT, Livesey MR, Zhao C, Gregory JM, James OT, Cleary EM, Chouhan AK, Gane AB, Perkins EM, Dando O et al. 2018. C9ORF72 repeat expansion causes vulnerability of motor neurons to Ca(2+)-permeable AMPA receptor-mediated excitotoxicity. *Nat Commun* **9**: 347.
Shannon CP, Chen V, Takhar M, Hollander Z, Balshaw R, McManus BM, Tebbutt SJ, Sin DD, Ng RT. 2016. SABRE: a method for assessing the stability of gene modules in complex tissues and subject populations. *BMC Bioinformatics* **17**: 460.

Wagner F, Yan Y, Yanai I. 2018. K-nearest neighbor smoothing for high-throughput single-cell RNA-Seq data. *bioRxiv* doi:https://doi.org/10.1101/217737.

Zhang Y, Sloan SA, Clarke LE, Caneda C, Plaza CA, Blumenthal PD, Vogel H, Steinberg GK, Edwards MS, Li G et al. 2016. Purification and Characterization of Progenitor and Mature Human Astrocytes Reveals Transcriptional and Functional Differences with Mouse. *Neuron* **89**: 37-53.
